# Proprioceptive Training for Knee Osteoarthritis: A Systematic Review and Meta-Analysis of Randomized Controlled Trials

**DOI:** 10.3389/fmed.2021.699921

**Published:** 2021-10-28

**Authors:** Yi Wang, Zugui Wu, Zehua Chen, Xiangling Ye, Guoqian Chen, Jiaman Yang, Peiming Zhang, Fang Xie, Yingxin Guan, Jiatao Wu, Weijian Chen, Zixuan Ye, Xuemeng Xu

**Affiliations:** ^1^The Fifth Clinical Medical College of Guangzhou University of Chinese Medicine, Guangzhou, China; ^2^Department of Orthopaedic Surgery, Zhejiang Provincial Hospital of Chinese Medicine, Hangzhou, China; ^3^Medical College of Acu-Moxi and Rehabilitation, Guangzhou University of Chinese Medicine, Guangzhou, China; ^4^Affiliated Changde Hospital, Hunan University of Traditional Chinese Medicine, Changde, China; ^5^Guangdong Second Traditional Chinese Medicine Hospital, Guangzhou, China

**Keywords:** proprioceptive training, knee osteoarthritis, rehabilitation, systematic review, meta-analysis

## Abstract

**Background:** There is increased interest in proprioceptive training for knee osteoarthritis (KOA). However, little consensus supports the effectiveness of this intervention.

**Objective:** This meta-analysis aimed to assess the effects of proprioceptive training on symptoms, function, and proprioception in people with KOA.

**Methods:** The PubMed, Cochrane Library, Web of Science, and EMBASE databases were systematically searched from the inception dates to April 16, 2021 for relevant randomized controlled trials (RCTs). Data were pooled by calculating the standardized mean differences (SMDs) and 95% confidence intervals (CIs). A random-effects model was used for the analyses.

**Results:** A total of 24 RCTs involving 1,275 participants were included in our analysis. This study indicated that compared to no intervention, proprioceptive training significantly improved pain, stiffness, physical function, joint position sense (JPS), muscle strength, mobility, and knee ROM (*P* < 0.05) in people with KOA. When compared to other non-proprioceptive training, proprioceptive training provided better results in terms of JPS (SMD = −1.28, 95%CI: [−1.64, −0.92], *I*^2^ = 0%, *P* < 0.00001) and mobility (timed walk over spongy surface) (SMD = −0.76, 95%CI: [−1.33, −0.18], *I*^2^ = 64%, *P* = 0.01), and other results are similar. When proprioceptive training plus other non-proprioceptive training compared to other non-proprioceptive training, the two groups showed similar outcomes, but there was a greater improvement for JPS (SMD = −1.54, 95%CI: [−2.74, −0.34], *I*^2^ = 79%, *P* = 0.01), physical function (SMD = −0.34, 95%CI: [−0.56, −0.12], *I*^2^ = 0%, *P* = 0.003), and knee ROM (*P* < 0.05) in the proprioceptive training plus other non-proprioceptive training group. When proprioceptive training plus conventional physiotherapy compared against conventional physiotherapy, the two groups demonstrated similar outcomes, but there was a significant improvement for JPS (SMD = −0.95, 95%CI: [−1.73, −0.18], *I*^2^ = 78%, *P* = 0.02) in the proprioceptive training plus conventional physiotherapy group.

**Conclusions:** Proprioceptive training is safe and effective in treating KOA. There is some evidence that proprioceptive training combined with general non-proprioceptive training or conventional physiotherapy appears to be more effective and should be considered as part of the rehabilitation program. However, given that the majority of current studies investigated the short-term effect of these proprioceptive training programs, more large-scale and well-designed studies with long-term follow up are needed to determine the long-term effects of these proprioceptive training regimes in KOA.

**Systematic Review Registration:** https://www.crd.york.ac.uk/prospero/#recordDetails, PROSPERO, identifier: CRD42021240587.

## Introduction

Osteoarthritis (OA) is a chronic, degenerative joint disease that mainly affects weight-bearing joints (1). Knee osteoarthritis (KOA) is the most common form of OA and affects ~265 million people worldwide (2). Additionally, it is one of the leading causes of disability (3). Due to population aging and the increasing incidence of obesity, the prevalence of KOA is rising (4), increasing the socioeconomic burden for affected individuals and healthcare systems (5, 6). KOA is clinically characterized by pain, joint stiffness, reduced joint motion, quadriceps weakness, and proprioceptive deficits (7, 8). Its pathology may be associated with degenerative lesions in cartilage secondary to inflammation linked to hyperplasia and chondrocyte apoptosis (9, 10). The treatment options for knee osteoarthritis include non-pharmacological, pharmacological, or surgical measures (11, 12). Current clinical guidelines recommend a multimodal, individualized non-pharmacological treatment program as first-line treatment for KOA (13, 14). The core treatment included therapeutic exercise (e.g., aerobic, resistance, strengthening, and proprioception), physical therapy (e.g., massage, ultrasound, and thermotherapy), lifestyle modifications, weight management, and education (11, 13, 15–17). These interventions aimed to relieve pain, improve physical function, and slow the progression of the disease (18–20).

Although the etiology of KOA remains largely undefined (21), some risk factors that have been proved to influence KOA susceptibility include age, sex, obesity, knee injury, muscle weakness, genetics, and ethnicity (22–26). Furthermore, many recent studies indicated that proprioceptive impairments could be an important risk factor for the incidence and progression of KOA (27–31). Proprioception mostly consists of several different biomechanical components, such as JPS, motion sense, velocity, and force (32). Proprioception derives from proprioceptors in skeletal muscles, tendons, ligaments, and joint capsule (33–35). With the progression of KOA, proprioception could also be decreased (30, 36). Additionally, proprioceptive impairments could be a cause of knee pain or activity limitations in KOA patients (29, 37).

The programs focused on improving or restoring proprioception have been referred to as proprioceptive trainings (38). Recently, although many studies have explored and analyzed the efficacy of proprioceptive training for KOA (39–41), several investigators have reported that there was insufficient clinical evidence of proprioceptive training for KOA, and the results of previous studies have been also inconsistent (35, 42). To our knowledge, two previous reviews have reported on the effects of proprioceptive training in KOA (35, 43). However, one of these is now about 10 years old, and the number of studies included is small (35), whereas the one recent review did not compare the efficacy of proprioceptive training with other general non-proprioceptive training for KOA (42). Besides, these two reviews did not assess the safety of proprioceptive training and investigate the effects of combinations of proprioceptive training with other interventions in KOA. Our interest in updating the current evidence has increased as the numerous renewals of high-quality studies on proprioceptive training in treating KOA (43–45). Therefore, we conducted this study to summarize all current high-quality evidence on the clinical efficacy and safety of proprioceptive training for KOA, and to provide a quantitative assessment. This will be very important and necessary, and the results of the study will provide evidence and guidance for the promotion and application of proprioceptive training in clinical practice.

## Methods

Methods and reporting of this systematic review and meta-analysis adhere to the Preferred Reporting Items for Systematic Reviews and Meta-Analyses (PRISMA) guidelines (46). The protocol for this meta-analysis was registered with PROSPERO (CRD 42021240587).

### Search Strategy

We systematically searched the PubMed, Cochrane Library, Web of Science, and EMBASE databases from the inception dates to April, 16 2021. The following search string and MeSH terms were used to search: “Proprioceptive training,” “Knee osteoarthritis,” and “Randomized Controlled Trial.” In addition, we also manually searched the reference lists of selected articles and reviews for additional relevant articles. Two independent reviewers (YW and ZW) screened eligible articles, all disagreements were resolved by independent third-party review and consensus. The search strategy is detailed in Supplementary Table S1.

### Selection Criteria

We developed eligibility criteria for this study using the (PICOS) description model (47) of participants, interventions, comparisons, outcomes, and study design.

### Participants

Inclusion criteria:

- Adult patients with KOA

Exclusion criteria:

- Participants who had suffered knee joint trauma or surgery
- Participants with other muscular, joint, or neurological conditions.

### Intervention

- Proprioceptive training

Proprioceptive training includes proprioceptive, balance, and sensorimotor training. However, no restrictions were made in terms of the frequency, duration, or intensity of the intervention. Additionally, we excluded studies where the intervention was whole-body vibration or water training.

### Comparators

- Proprioceptive training vs. no intervention
- Proprioceptive training vs. other non-proprioceptive training (e.g., resistance and strength training)
- Proprioceptive training with other non-proprioceptive training vs. other non-proprioceptive training
- Proprioceptive training with conventional physiotherapy vs. conventional physiotherapy.

### Outcomes

Primary outcomes:

- Pain [visual analog scale (VAS), numeric rating scale (NRS), Western Ontario and McMaster Universities Osteoarthritis Index (WOMAC) scale, Knee Injury and Osteoarthritis Outcome Score (KOOS)]
- Stiffness (WOMAC, KOOS)
- Physical function (WOMAC, KOOS)

Secondary outcomes:

- Joint position sense (JPS)
- Muscle strength
- Mobility [walking-speed timed test (WST), Get up and go (GUG) test]
- Knee range of motion (ROM)
- Balance
- Adverse events.

### Study Design

- Randomized controlled trials (RCTs)
- Published in English.

### Data Extraction

Two independent reviewers (YW and ZW) used a standardized form extract the following information from each study in accordance with the pre-specified study protocol, including participant characteristics (e.g., age, gender, stage of KOA), study characteristics (e.g., lead author, publication year, country of origin, intervention frequency and duration, follow-up period), and main outcomes. Disagreements were resolved by discussion and consensus between the reviewers. If necessary, we will contact the corresponding authors to obtain the required information.

### Quality Assessment

The methodological quality of each included studies was assessed independently by two reviewers (YW and ZW) using the Physiotherapy Evidence Database (PEDro) scale (46, 48). The PEDro scale (range, 0–10, with 10 indicating highest quality) is a reliable and valid appraisal tool to assess the quality of physiotherapy-based RCTs (48, 49). Furthermore, we also assessed the overall quality of the evidence for each outcome through the Grades of Recommendations, Assessment, Development, and Evaluation (GRADE) approach (47). Any inconsistency was resolved through independent third-party review and consensus.

### Statistical Analysis

Data analyses were performed using Revman (version 5.3, Cochrane Collaboration) and Stata (version 13.0). We converted other forms of data (i.e., median, mean [95%CI], standard error and interquartile range) to means (SDs) based on the Cochrane Handbook for Systematic Reviews of Interventions (50). The standard mean differences (SMDs) with 95% confidence intervals (95% CIs) for pooled data were calculated. We pooled the data using a random-effects model and examined statistical heterogeneity by calculating the *I*^2^ statistic. An *I*^2^ statistic >50% was considered to be substantially heterogeneous (50, 51). We planned to perform subgroup analyses to identify potential determinants of efficacy. Sensitivity analysis was also used to explore potential sources of heterogeneity between studies and to assess whether the significant results were robust. Furthermore, we evaluated publication bias by examining funnel plots and statistical asymmetry tests (Begg's test and Egger's regression). *P* < 0.05 was considered statistically significant.

## Results

### Study Selection

**Figure 1:**
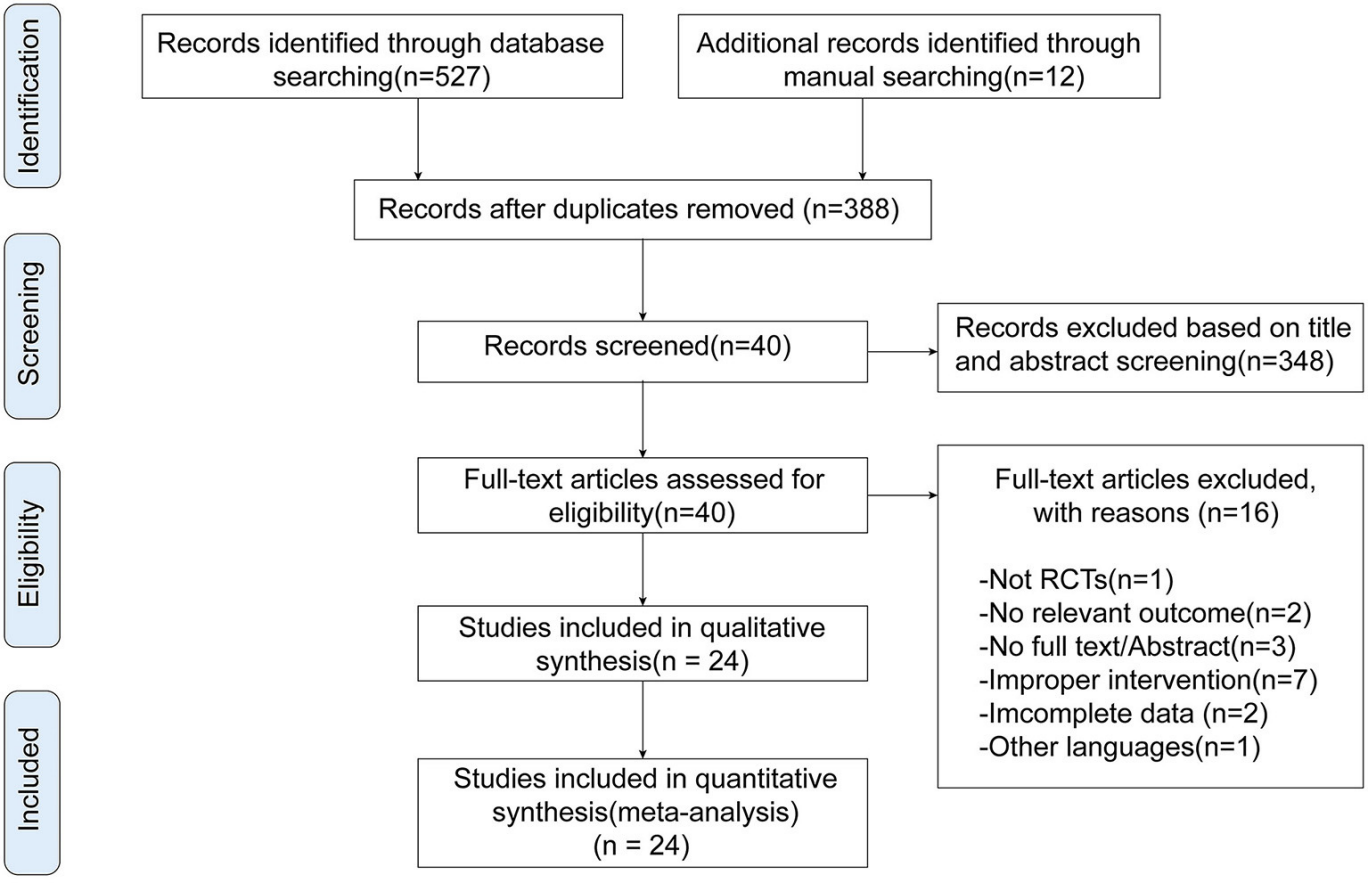
Flowchart of study selection.

We retrieved 539 potentially relevant records through electronic and manual searching. EndNote X9 (Bld 12062) was used to screen eligible studies. Initially, 388 articles remained after removal of duplicates. Then we selected 40 articles for full-text review after screening their titles and abstracts. Fourteen articles were excluded since they did not meet inclusion criteria (e.g., non-randomization, no relevant outcome, ineligible intervention, non-English); two studies did not provide complete data for calculation of effect sizes. Ultimately, we selected 24 studies (39–41, 43–45, 52–69) for inclusion in our study (Figure 1).

### Study Characteristics

Twenty-four studies involving a total of 1,275 participants with KOA were included. The studies were from Brazil (52), Turkey (60, 64, 66, 69), Australia (58), Canada (59), Thailand (62), Egypt (55, 65), Iran (44), Malaysia (63), the United States (40, 41, 61), India (39, 43, 54, 68), and China (45, 53, 56, 57, 67). The publication intervals of 24 included articles were from 2005 to 2020, and the sample size ranging from 15 to 183 participants. Twenty-two studies reported the age of the participants. Their mean (SD) age ranged from 40.87 (4.91) to 72.4 (11.02) years. Except for three studies (39, 54, 61) that did not specify diagnostic criteria, all other studies used the Kellgren and Lawrence (70) radiological assessment scale or the American College of Rheumatologist's diagnostic classification (71) to diagnose KOA. Regarding the comparison of interventions, seven studies compared proprioceptive training to no intervention (40, 45, 56, 57, 59, 60, 67), eight studies compared proprioceptive training to other non-proprioceptive training (40, 52, 56, 57, 61, 62, 67, 68), seven studies compared proprioceptive training plus other non-proprioceptive training to other non-proprioceptive training (40, 41, 55, 58, 64–66), and seven studies compared proprioceptive training plus conventional physiotherapy to conventional physiotherapy (39, 43, 44, 53, 54, 63, 69).

The duration of the intervention ranges from 2 to 16 weeks. In addition, the shortest follow-up period was only 2 weeks (63) and the longest was 52 weeks (41, 66). The characteristics of the included studies are summarized in Table 1. And the detailed descriptions of interventions are presented in Supplementary Table S2.

**Table 1:** Characteristics of the included studies.

**References: publication year, country**	**Study design**	**Participant characteristics**	**Diagnosis criteria/K-L grade**	**Intervention**	**Intervention characteristics, follow-up**	**Outcome measures**	**PEDro score**
Gomiero et al. (52), 2018, Brazil	RCT	No. (M/F): 64 (3/61) IG: 32(2/30) CG: 32 (1/31) Dropout: 2 Mean Age ± SD, years: IG: 61.6 ± 6.8 CG: 61.8 ± 6.4	ACR/K-L I-IV	IG: Proprioceptive training CG: Resistance training	16 weeks, 2 sessions/week	VAS; WOMAC (total); Mobility; Balance	8
Tsauo et al. (53), 2008, Taiwan, China	RCT	No. (M/F):29 (5/24) IG: 15(1/14) CG: 14 (4/10) Dropout: 31 Mean Age ± SD, years: IG: 61.7 ± 6.6 CG: 60.1 ± 6.7	ACR/K-L II-III	IG: Proprioceptive training plus Conventional physiotherapy CG: Conventional physiotherapy	8 weeks, 3 sessions/week, 30 min/session	WOMAC (pain); WOMAC (stiffness); WOMAC (physical function); JPS	5
Kumar et al. (54), 2013, India	RCT	No. (M/F): 44 (19/25) IG: 22 CG: 22 Dropout: 0 Mean Age ± SD, years: IG: 53.18 ± 6.88 CG: 53.32 ± 5.36	NA	IG: Proprioceptive training plus Conventional physiotherapy CG: Conventional physiotherapy	4 weeks, 3 sessions/week	NRS; WOMAC (physical function); JPS	6
Fitzgerald et al. (41), 2011, USA	RCT	No. (M/F): 183 (61/122) IG: 91 (31/60) CG: 92 (30/62) Dropout: 38 Mean Age ± SD, years: IG: 63.3 ± 8.9 CG: 64.6 ± 8.4	ACR/K-L II-IV	IG: Proprioceptive training plus Strength training CG: Strength training	8, 26, and 52 weeks, 3 sessions/week, ≥30 min/session	NRS; WOMAC (physical function); WOMAC (total); Mobility	7
Rogers et al. (40), 2012, USA	RCT	No. (M/F):33 (13/20) IG (PT): 8 CG (RT): 8 CG (PT + RT): 9 CG: 8 Dropout: 11 Mean Age ± SD, years: IG (PT): 70.7 ± 10.7 CG (RT): 70.8 ± 6.5 CG (PT + RT): 68.8 ± 10.1 CG: 71.2 ± 10.9	ACR	IG (PT): Proprioceptive training CG (RT): Resistance training CG (PT + RT): Proprioceptive training plus Resistance training CG: Without intervention	8 weeks, 3 sessions/week, 30–40 min/session	WOMAC (pain); WOMAC (stiffness); WOMAC (physical function); WOMAC (total)	6
Ahmed et al. (55), 2011, Egypt	RCT	No. (M/F): 40 IG: 20 CG: 20 Dropout: 0 Mean Age ± SD, years: IG: 60 ± 3.6 CG: 62 ± 3.2	ACR/K-L II	IG: Proprioceptive training plus Traditional exercise CG: Traditional exercise	6 weeks, 3 sessions/week	VAS; JPS	7
Lin et al. (56), 2007, Taiwan, China	RCT	No. (M/F):81 (19/62) IG (PT): 29(9/20) CG (RT): 26(5/21) CG: 26(5/21) Dropout: 8 Mean Age ± SD, years: IG (PT): 61.6 ± 8.1 CG (RT): 61.0 ± 7.7 CG: 62.8 ± 6.3	ACR/K-L I-III	IG (PT): Proprioceptive training CG (RT): Resistance training CG: Without intervention	8 weeks, 3 sessions/week	Muscle strength	5
Jahanjoo et al. (44), 2019, Iran	RCT	No. (M/F):60 (13/47) IG: 30(8/22) CG: 30(5/25) Dropout: 0 Mean Age ± SD, years: IG: 57.53 ± 0.8 CG: 55.57 ± 1.6	ACR/K-L I-III	IG: Proprioceptive training plus Conventional physiotherapy CG: Conventional physiotherapy	5 weeks, 2 sessions/week	VAS; WOMAC (pain); WOMAC (stiffness); WOMAC (physical function); WOMAC (total); Mobility	7
Lin et al. (57), 2009, Taiwan, China	RCT	No. (M/F): 108 (33/75) IG (PT): 36 (11/25) CG (ST): 36 (12/24) CG: 36 (10/26) Dropout: 5 Mean Age ± SD, years: IG (PT): 63.7 ± 8.2 CG (ST): 61.6 ± 7.2 CG: 62.2 ± 6.7	K-L I-III	IG (PT): Proprioceptive training CG (ST): Strength training CG: Without intervention	8 weeks, 3 sessions/week	WOMAC (pain); WOMAC (physical function); JPS; Mobility	8
Gohil and Shukla (43), 2020, India	RCT	No. (M/F):22 IG: 11 CG:11 Dropout: 0	K-L II-III	IG: Proprioceptive training plus Conventional physiotherapy CG: Conventional physiotherapy	8 weeks, 3 sessions/week	NRS; WOMAC (physical function); JPS	4
Kirthika et al. (39), 2018, India	RCT	No. (M/F):40 IG: 20 CG:20 Dropout: 3 Mean Age ± SD, years: IG: 58.2 ± 5.7 CG: 58.8 ± 5.3	NA	IG: Proprioceptive training plus Conventional physiotherapy CG: Conventional physiotherapy	12 weeks, 5 sessions/week	VAS; WOMAC (total)	6
Pazit et al. (58), 2018, Australia	RCT	No. (M/F):19 (9/10) IG: 10(4/6) CG: 9(5/4) Dropout: 0 Mean Age ± SD, years: IG: 65.10 ± 4.77 CG: 67.78 ± 6.28	ACR	IG: Proprioceptive training plus Resistance training CG: Resistance training	8 weeks, 2 sessions/week	WOMAC (pain); WOMAC (stiffness); WOMAC (physical function); WOMAC (total); Mobility	8
Takacs et al. (59), 2017, Canada	RCT	No. (M/F):36(6/30) IG: 17(0/17) CG: 19(6/13) Dropout: 4 Mean Age ± SD, years: IG: 66.1 ± 8.7 CG: 67.1 ± 5.4	ACR/K-L II-IV	IG: Proprioceptive training CG: Without intervention	10 weeks, 4 sessions/week	NRS; WOMAC (physical function); Muscle strength	7
Sekir and Gür (60), 2005, Turkey	RCT	No. (M/F):22(6/16) IG: 12(3/9) CG: 10(3/7) Dropout: 0 Mean Age ± SD, years: IG: 59 ± 8.9 CG: 62 ± 8.1	ACR/K-L II-III	IG: Proprioceptive training CG: Without intervention	6 weeks, 2 sessions/week	VAS; JPS; Muscle strength	6
Rogers et al. (61), 2011, USA	RCT	No. (M/F):15 IG: 6 CG: 9 Dropout: 5 Mean Age ± SD, years: IG: 69.29 ± 11.36 CG: 72.4 ± 11.02	NA	IG: Proprioceptive training CG: Strength training	8 weeks, 3 sessions/week, 30 min/session	WOMAC (pain); WOMAC (stiffness); WOMAC (physical function); Mobility	5
Chaipinyo and Karoonsupcharoen (62), 2009, Thailand	RCT	No. (M/F):48(11/31) IG: 24(9/15) CG: 24(2/22) Dropout: 6 Mean Age ± SD, years: IG: 62 ± 6 CG: 70 ± 6	ACR	IG: Proprioceptive training CG: Strength training	4 weeks, 5 sessions/week	KOOS (pain); KOOS (symptoms); Muscle strength; Mobility	6
Mondam et al. (63), 2012, Malaysia	RCT	No. (M/F):50 IG: 25 CG:25 Dropout: 0	K-L I-II	IG: Proprioceptive training plus Conventional physiotherapy CG: Conventional physiotherapy	2 weeks, 7 sessions/week	VAS; WOMAC (total); Knee ROM	6
Diracoglu et al. (64), 2005, Turkey	RCT	No. (M/F):66(0/66) IG: 32 CG: 28 Dropout: 6 Mean Age ± SD, years: IG: 50.3 ± 6.5 CG: 50.8 ± 7.9	ACR/K-L I-II	IG: Proprioceptive training plus Strength training CG: Strength training	8 weeks, 3 sessions/week	WOMAC (physical function)	6
Elgendy et al. (65), 2005, Egypt	RCT	No. (M/F):30 IG: 15 CG:15 Dropout: 0	ACR	IG: Proprioceptive training plus Traditional exercise CG: Traditional exercise	8 weeks, 3 sessions/week	VAS; JPS	6
Song et al. (45), 2020, China	RCT	No. (M/F):29(11/18) IG: 13(5/8) CG: 16(6/10) Dropout: 7 Mean Age ± SD, years: IG: 68.5 ± 4.3 CG: 67.4 ± 3.4	K-L I-III	IG: Proprioceptive training CG: Without intervention	12 weeks, 3 sessions/week, 60 min/session	WOMAC (pain); Knee ROM	4
Diracoglu et al. (66), 2008, Turkey	RCT	No. (M/F):66(0/66) IG: 32 CG: 28 Dropout: 6 Mean Age ± SD, years: IG: 50.3 ± 6.5 CG: 50.8 ± 7.9	ACR/K-L I-II	IG: Proprioceptive training plus Strength training CG: Strength training	8.52 weeks, 3 sessions/week	WOMAC (pain); WOMAC (stiffness); WOMAC (physical function); WOMAC (total)	6
Jan et al. (67), 2009, Taiwan, China	RCT	No. (M/F):106(0/66) IG(PT): 36(12/24) CG(RT): 35(10/25) CG:35(11/24) Dropout: 6 Mean Age ± SD, years: IG(PT): 62.0 ± 6.7 CG(RT): 63.2 ± 6.8 CG:62.2 ± 6.7	ACR/K-L I-III	IG (PT): Proprioceptive training CG (RT): Resistance training CG: Without intervention	8 weeks, 3 sessions/week	WOMAC (physical function); JPS; Muscle strength; Mobility	7
Vamsidhar et al. (68), 2017, India	RCT	No. (M/F):30 IG: 15 CG: 15 Dropout: 0 Mean Age ± SD, years: IG: 40.87 ± 4.91 CG: 41.53 ± 6.28	K-L II-III	IG: Proprioceptive training CG: Strength training	3 weeks, 3 sessions/week	VAS; WOMAC (total)	6
Duman et al. (69), 2012, Turkey	RCT	No. (M/F): 54 (5/49) IG: 30 CG: 24 Dropout: 0 Mean Age ± SD, years: 64 ± 3.7	ACR/K-L III-IV	IG: Proprioceptive training plus Conventional physiotherapy plus NSAID meloxicam CG: Conventional physiotherapy plus NSAID meloxicam	3 weeks, 5 sessions/week	WOMAC (pain); WOMAC (stiffness); WOMAC (physical function); WOMAC (total); JPS	6

*ACR, American College of Rheumatologists; CG, control group; CP, conventional physiotherapy; F, female; IG, intervention group; JPS, joint position sense; K-L, Kellgren and Lawrence radiological assessment scale; KOOS, Knee Injury and Osteoarthritis Outcome Score; M, male; NRS, numeric rating scale; NSAID, non-steroidal anti-inflammatory drug; PEDro, Physiotherapy Evidence Database; PT, proprioceptive training; RCT, Randomized Controlled Trials; ROM, range of motion; RT, resistance training; SD, Standard deviation; ST, strength exercise; TE, traditional exercise; VAS, visual analog scale; WOMAC, Western Ontario and McMaster Universities Osteoarthritis Index*.

### Quality of Studies

The mean PEDro scale score for all studies was 6.25 (range, 4–8; Table 2), suggesting that the studies were of moderate quality. All 24 studies satisfied four of the PEDro criteria, namely “random allocation,” “similar baseline,” “between-group statistics,” and “point measures,” but only eight studies (40, 41, 52, 57–59, 61, 62) used concealed allocation to minimize allocation bias. However, except for two (29, 38), the remaining studies did not account for “the blinding of the subjects and therapists,” 12 (39, 41, 44, 52, 53, 55, 57–59, 64, 66, 67) of the studies employed assessor blinding. In addition, six of the studies (40, 41, 43, 45, 53, 61) lost more than 15% participants during follow-up and the inconsistent use of “intention-to-treat” analyses were found to be consistent trial limitations in most of the studies.

**Table 2:** PEDro scores of the included studies.

**Study**	**Year**	**Eligibility criteria**	**Random allocation**	**Concealed allocation**	**Similar baseline**	**Blinding subjects**	**Blinding therapists**	**Blinding assessors**	**Dropout <15%**	**Intention to treat**	**Between-group statistics**	**Point measures**	**Total score**
Gomiero et al. (52)	2018	Yes	Yes	Yes	Yes	No	No	Yes	Yes	Yes	Yes	Yes	8
Tsauo et al. (53)	2008	Yes	Yes	No	Yes	No	No	Yes	No	No	Yes	Yes	5
Rogers et al. (40)	2012	Yes	Yes	Yes	Yes	Yes	No	No	No	No	Yes	Yes	6
Sekir and Gür (60)	2005	Yes	Yes	No	Yes	No	No	No	Yes	Yes	Yes	Yes	6
Rogers et al. (61)	2011	Yes	Yes	Yes	Yes	No	No	No	No	No	Yes	Yes	5
Pazit et al. (58)	2018	Yes	Yes	Yes	Yes	No	No	Yes	Yes	Yes	Yes	Yes	8
Takacs et al. (59)	2017	Yes	Yes	Yes	Yes	No	No	Yes	Yes	No	Yes	Yes	7
Lin et al. (56)	2007	Yes	Yes	No	Yes	No	No	No	Yes	No	Yes	Yes	5
Lin et al. (57)	2009	Yes	Yes	Yes	Yes	No	No	Yes	Yes	Yes	Yes	Yes	8
Jahanjoo et al. (44)	2019	Yes	Yes	No	Yes	No	No	Yes	Yes	Yes	Yes	Yes	7
Chaipinyo and Karoonsupcharoen (62)	2009	Yes	Yes	Yes	Yes	No	No	No	Yes	No	Yes	Yes	6
Fitzgerald et al. (41)	2011	Yes	Yes	Yes	Yes	No	No	Yes	No	Yes	Yes	Yes	7
Duman et al. (69)	2012	Yes	Yes	No	Yes	No	No	No	Yes	Yes	Yes	Yes	6
Diracoglu et al. (64)	2005	Yes	Yes	No	Yes	No	No	Yes	Yes	No	Yes	Yes	6
Diracoglu et al. (66)	2008	Yes	Yes	No	Yes	No	No	Yes	Yes	No	Yes	Yes	6
Ahmed (55)	2011	Yes	Yes	No	Yes	No	No	Yes	Yes	Yes	Yes	Yes	7
Kumar et al. (54)	2013	Yes	Yes	No	Yes	No	No	No	Yes	Yes	Yes	Yes	6
Elgendy et al. (65)	2005	Yes	Yes	No	Yes	No	No	No	Yes	Yes	Yes	Yes	6
Gohil and Shukla (43)	2020	Yes	Yes	No	Yes	No	No	No	No	No	Yes	Yes	4
Song et al. (45)	2020	Yes	Yes	No	Yes	No	No	No	No	No	Yes	Yes	4
Kirthika et al. (39)	2018	Yes	Yes	No	Yes	No	Yes	Yes	Yes	Yes	Yes	Yes	8
Mondam et al. (63)	2012	Yes	Yes	No	Yes	No	No	No	Yes	Yes	Yes	Yes	6
Jan et al. (67)	2009	Yes	Yes	No	Yes	No	No	Yes	Yes	Yes	Yes	Yes	7
Vamsidhar et al. (68)	2017	Yes	Yes	No	Yes	No	No	No	Yes	Yes	Yes	Yes	6

*PEDro, Physiotherapy Evidence Database scale*.

### Assessed Outcomes and Evidence Synthesis

#### Primary Outcomes

##### Pain

**Figure 2:**
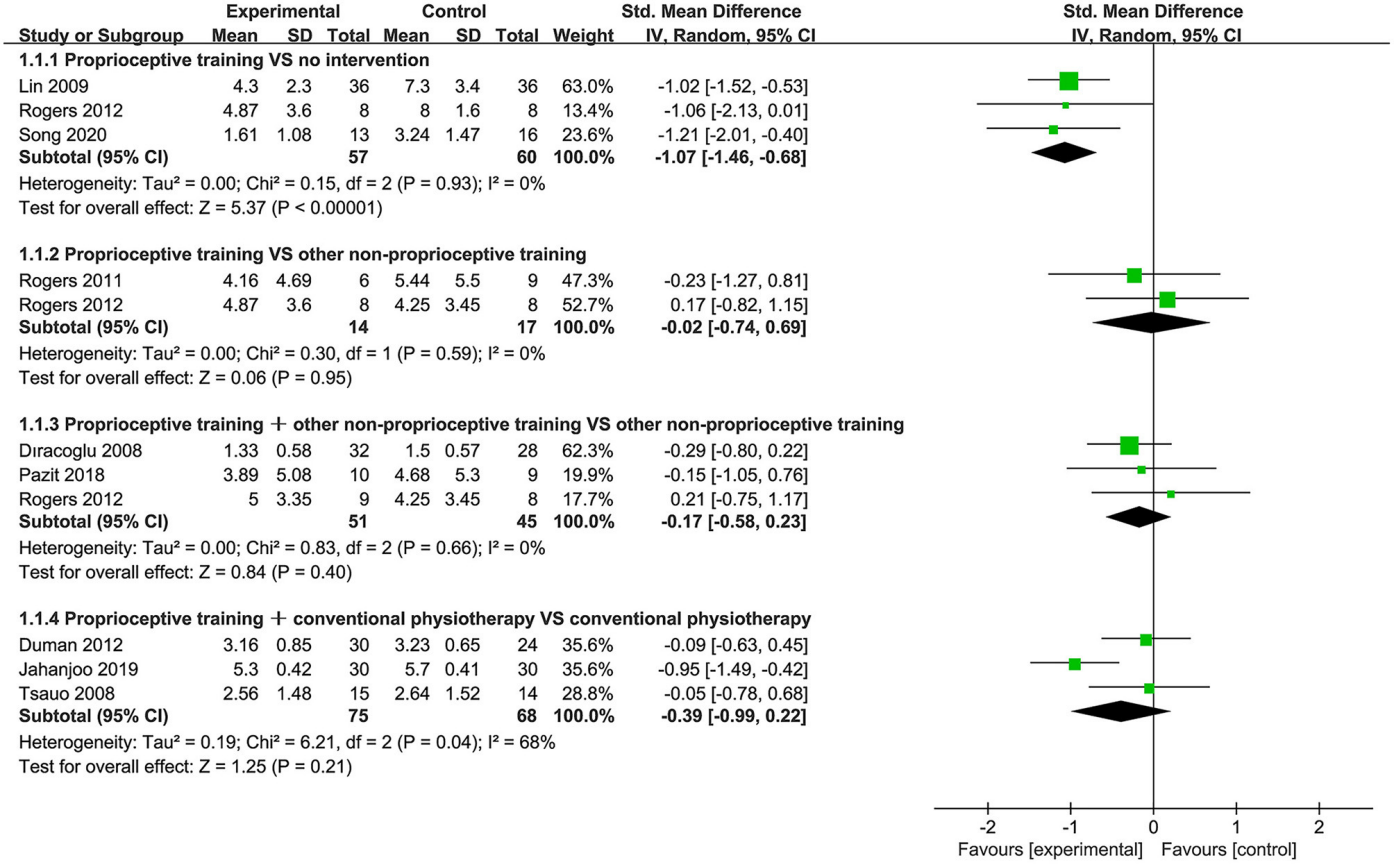
Forest plot of meta-analysis on pain.

Nine studies (40, 44, 45, 53, 57, 58, 61, 66, 69) used the WOMAC pain subscale to assess pain. Subgroup analysis showed significant improvement in pain for proprioceptive training compared to no intervention (SMD = −1.07, 95%CI: [−1.46, −0.68], *I*^2^ = 0%, *P* < 0.00001). When proprioceptive training compared against other non-proprioceptive training, the meta-analysis showed no statistically significant difference in alleviating pain (SMD = −0.02, 95%CI: [−0.74, 0.69], *I*^2^ = 0%, *P* = 0.95). When proprioceptive training plus other non-proprioceptive training compared to other non-proprioceptive training, the meta-analysis revealed no significant difference (SMD = −0.17, 95%CI: [−0.58, 0.23], *I*^2^ = 0%, *P* = 0.40). Additionally, the meta-analysis also showed no significant difference in relieving pain when proprioceptive training plus conventional physiotherapy compared against conventional physiotherapy (SMD = −0.39, 95%CI: [−0.99, 0.22], *I*^2^ = 68%, *P* = 0.21; Figure 2).

##### Stiffness

**Figure 3:**
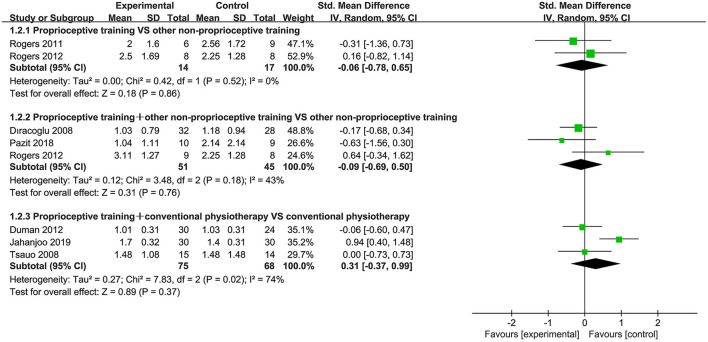
Forest plot of meta-analysis on stiffness.

Seven studies (40, 44, 53, 58, 61, 66, 69) used the WOMAC stiffness subscale to assess stiffness. When proprioceptive training compared to no intervention, only one study reported WOMAC stiffness score, and demonstrated that proprioceptive training effectively improved stiffness (*P* < 0.05). When proprioceptive training compared to other non-proprioceptive training, the subgroup analysis showed no statistically significant difference in improving stiffness (SMD = −0.06, 95%CI: [−0.78, 0.65], *I*^2^ = 0%, *P* = 0.86). When proprioceptive training plus other non-proprioceptive training compared against other non-proprioceptive training, the meta-analysis revealed no significant difference (SMD = −0.09, 95%CI: [−0.69, 0.50], *I*^2^ = 43%, *P* = 0.76). Furthermore, the meta-analysis also showed no significant difference in improving stiffness when proprioceptive training plus conventional physiotherapy compared against conventional physiotherapy (SMD = 0.31, 95%CI: [−0.37, 0.99], *I*^2^ = 74%, *P* = 0.37; Figure 3).

##### Physical Function

**Figure 4:**
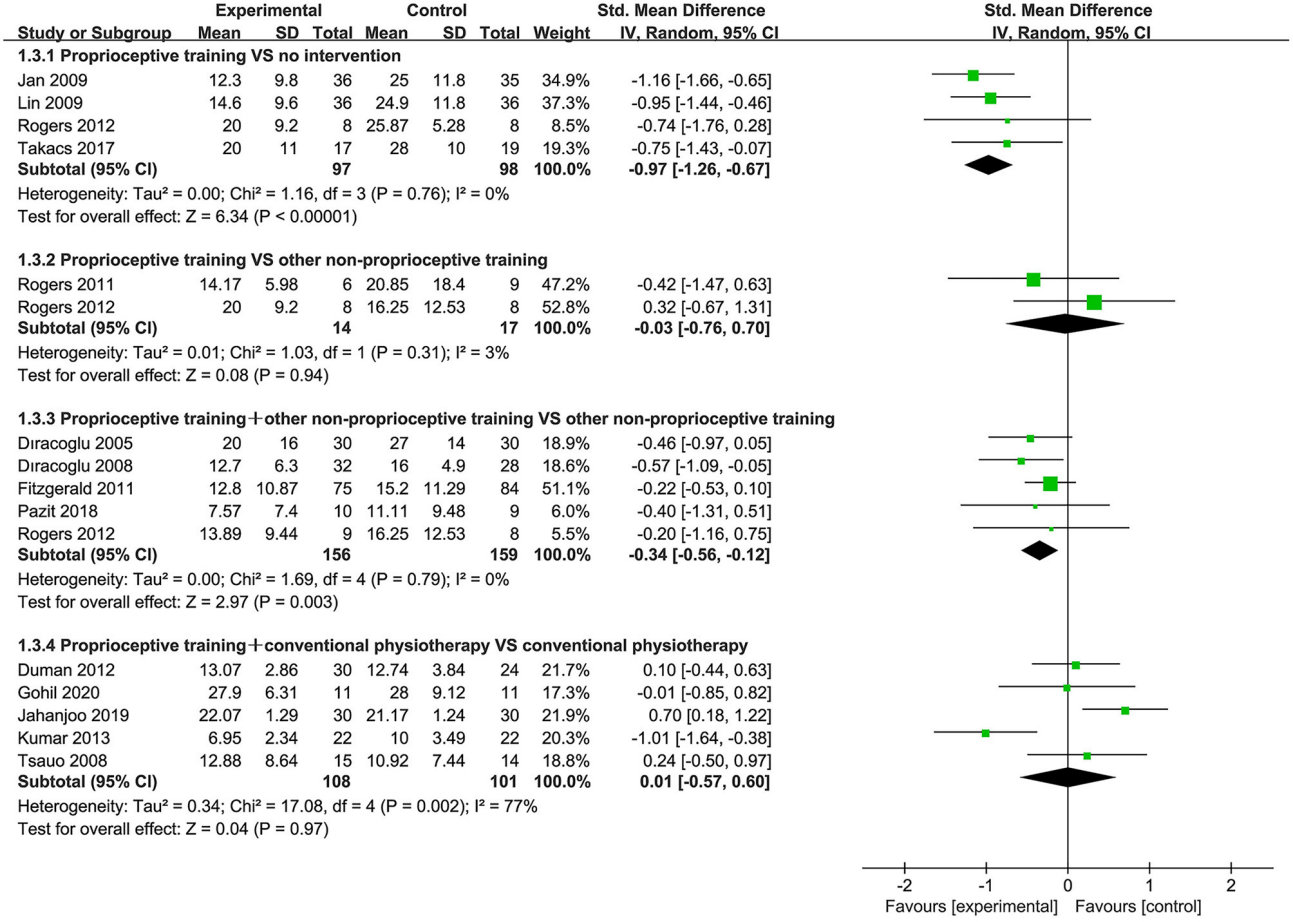
Forest plot of meta-analysis on WOMAC (physical function) score.

**Figure 5:**
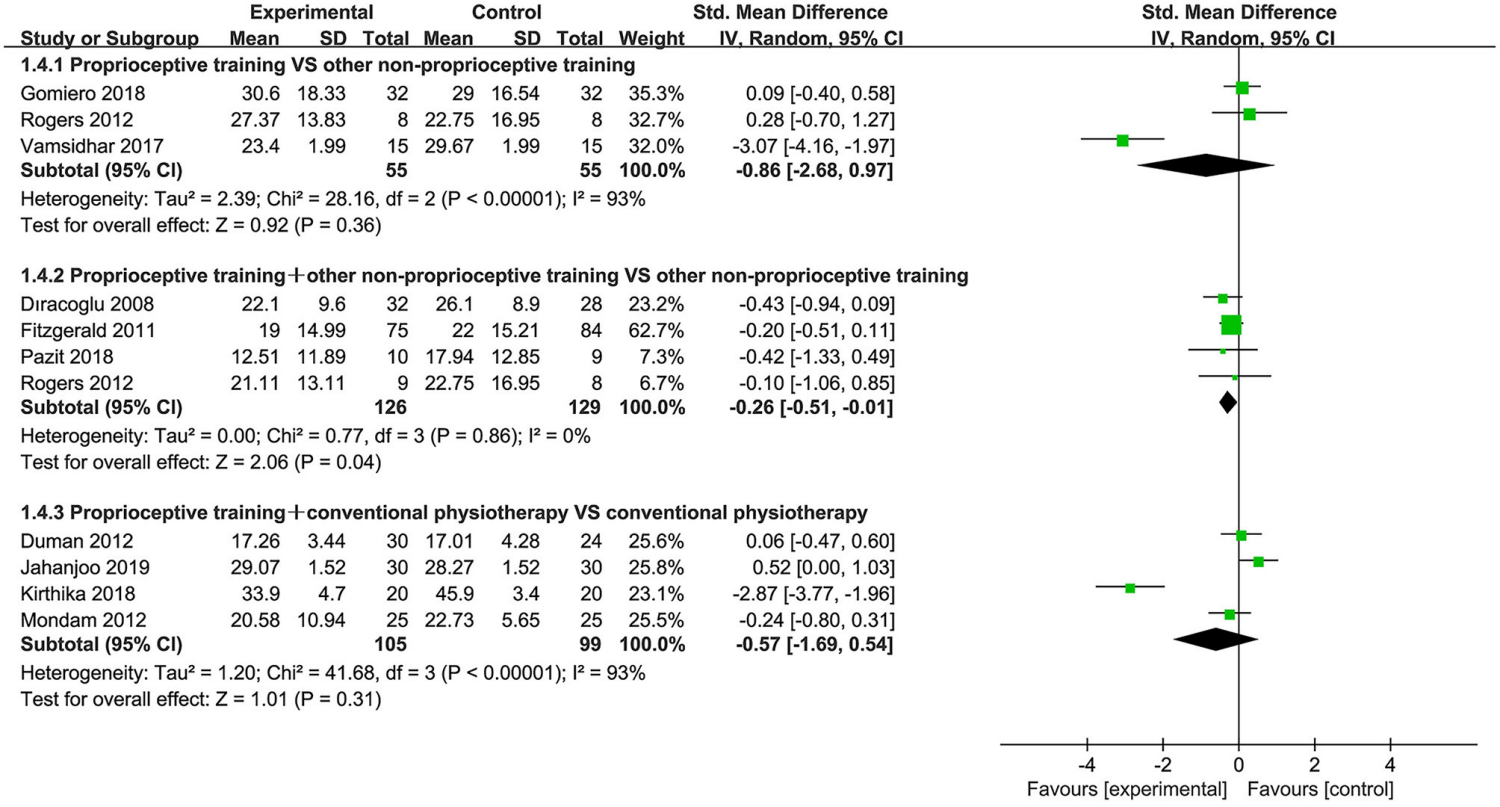
Forest plot of meta-analysis on WOMAC (total) score.

Eighteen studies (39–41, 43, 44, 52–54, 57–59, 61, 63, 64, 66–69) used the WOMAC physical function subscale and WOMAC total score to assess physical function. When proprioceptive training compared to no intervention, the subgroup analysis showed significant improvement in physical function (SMD = −0.97, 95%CI: [−1.26, −0.67], *I*^2^ = 0%, *P* < 0.00001). Also, one study (40) showed statistically significant difference in WOMAC total score (*P* < 0.05). When proprioceptive training compared to other non-proprioceptive training, the meta-analysis showed no statistically significant difference in improving physical function (SMD = −0.03, 95%CI: [−0.75, 0.69], *I*^2^ = 3%, *P* = 0.94). With respect to WOMAC total score, the meta-analysis also revealed no significant difference (SMD = −0.86, 95%CI: [−2.68, 0.97], *I*^2^ = 93%, *P* = 0.36). When proprioceptive training plus other non-proprioceptive training compared against other non-proprioceptive training, the meta-analysis revealed significant improvement in physical function (SMD = −0.34, 95%CI: [−0.56, −0.12], *I*^2^ = 0%, *P* = 0.003). Similarly, the meta-analysis showed significant difference in WOMAC total score (SMD = −0.26, 95%CI: [−0.51, −0.01], *I*^2^ = 0%, *P* = 0.04). Furthermore, the meta-analysis showed no significant difference in improving physical function when proprioceptive training plus conventional physiotherapy compared against conventional physiotherapy (SMD = 0.01, 95%CI: [−0.57, 0.60], *I*^2^ = 77%, *P* = 0.97). Similarly, the meta-analysis also showed no significant difference in WOMAC total score (SMD = −0.57, 95%CI: [−1.69, 0.54], *I*^2^ = 93%, *P* = 0.31; Figures 4, 5).

#### Secondary Outcomes

##### JPS

**Figure 6:**
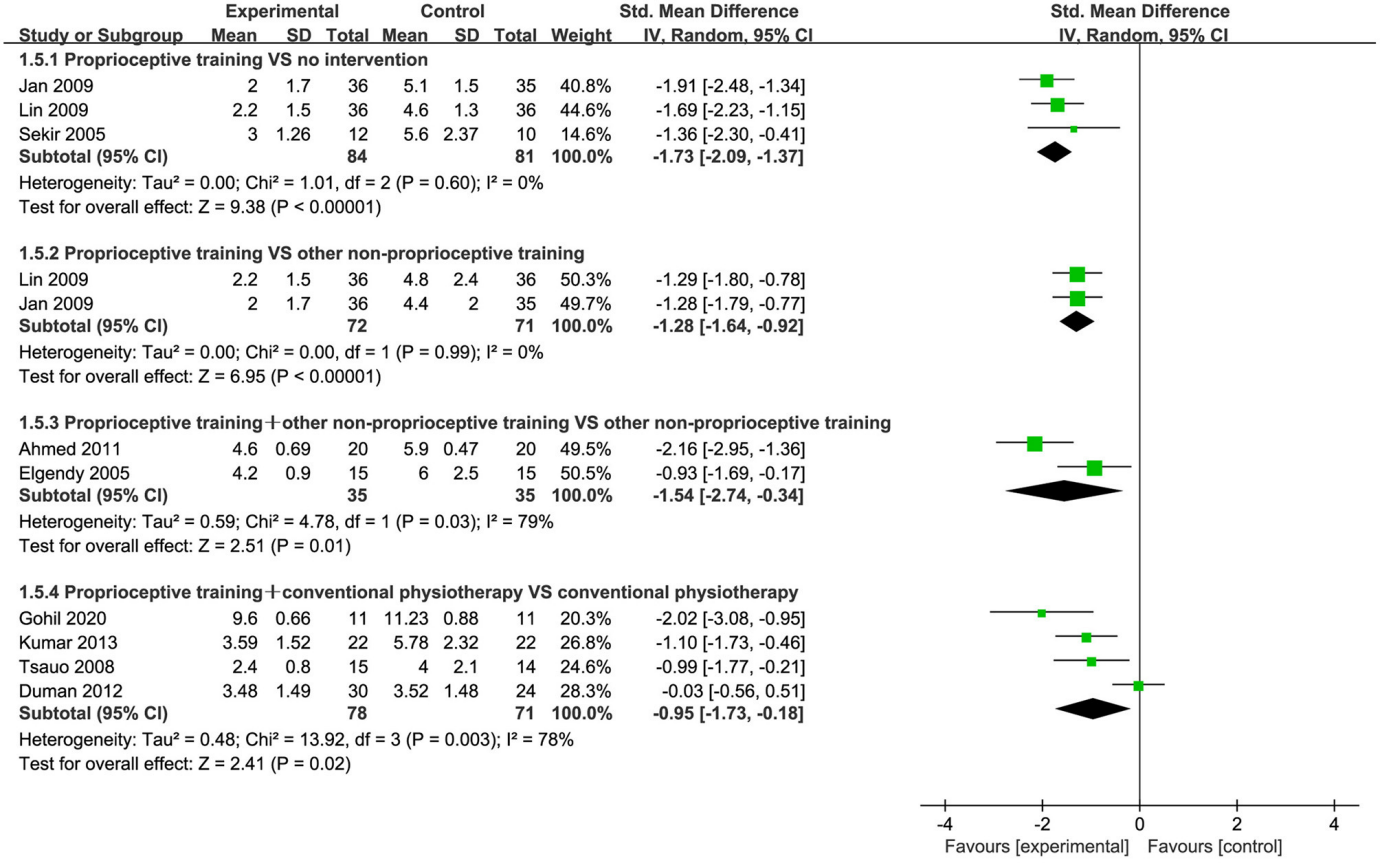
Forest plot of meta-analysis on JPS.

Nine studies (43, 53–55, 57, 60, 65, 67, 69) used the reposition error test to assess JPS. Subgroup analysis showed significant improvement in JPS for proprioceptive training compared to no intervention (SMD = −1.73, 95%CI: [−2.09, −1.37], *I*^2^ = 0%, *P* < 0.00001). When proprioceptive training compared to other non-proprioceptive training, the meta-analysis showed significant difference in improving JPS (SMD = −1.28, 95%CI: [−1.64, −0.92], *I*^2^ = 0%, *P* < 0.00001). When proprioceptive training plus other non-proprioceptive training compared against other non-proprioceptive training, the meta-analysis revealed statistically significant difference (SMD = −1.54, 95%CI: [−2.74, −0.34], *I*^2^ = 79%, *P* = 0.01). Additionally, the meta-analysis also showed significant improvement in JPS when proprioceptive training plus conventional physiotherapy compared against conventional physiotherapy (SMD = −0.95, 95%CI: [−1.73, −0.18], *I*^2^ = 78%, *P* = 0.02; Figure 6).

##### Muscle Strength

Three studies (56, 62, 67) assessed muscle strength by measuring knee flexion and extension torque. When proprioceptive training compared to no intervention, the subgroup analysis revealed that participants in the proprioceptive training group presented significantly increased knee flexion torque at the velocity of 60°/s (SMD = 0.65, 95%CI: [0.29, 1.01], *I*^2^ = 0%, *P* = 0.0004), but there was no significant difference at the velocity of 120 or 180°/s (*P* > 0.05; Table 3). Likewise, there was also significant greater improvement of knee extension torque in participants of the proprioceptive training group at the velocity of 60°/s (SMD = 0.42, 95%CI: [0.07, 0.78], *I*^2^ = 0%, *P* = 0.02), but no significant difference at the velocity of 120 or 180°/s (*P* > 0.05). When proprioceptive training compared against other non-proprioceptive training, the meta-analysis indicated that participants in the proprioceptive training group showed statistically significantly greater improvements in knee flexion torque at the velocity of 60°/s (SMD = 0.71, 95%CI: [0.30, 1.12], *I*^2^ = 0%, *P* = 0.0008; Table 4). Furthermore, Lin et al. (56) demonstrated that participants in the other non-proprioceptive training group presented significantly increased knee flexion torque at the velocity of 120 or 180°/s (*P* < 0.05). Nevertheless, the meta-analysis showed no statistically significant difference between the groups (SMD = 0.50, 95%CI: [−0.61, 1.61], *I*^2^ = 86%, *P* = 0.38) when knee extension torque was measured at the velocity of 60°/s. As in flexion, Lin et al. (56) indicated that participants in the other non-proprioceptive training group showed significantly higher knee flexion torque at the velocity of 120 or 180°/s (*P* < 0.05).

**Table 3:** Meta-analysis results of muscle strength and mobility when proprioceptive training compared to no intervention.

**Outcomes**	**IG (sample size)**	**CG (sample size)**	**SMD (95% CI)**	***P*-value**	***I*^**2**^ (%)**
Knee flexion torque at 60°/s	65	61	0.65 (0.29, 1.01)	0.0004	0
Knee flexion torque at 120°/s	65	61	0.32 (−0.03, 0.67)	0.08	0
Knee flexion torque at 180°/s	65	61	0.25 (−0.10, 0.60)	0.17	0
Knee extension torque at 60°/s	65	61	0.42 (0.07, 0.78)	0.02	0
Knee extension torque at 120°/s	65	61	0.30 (−0.05, 0.65)	0.09	0
Knee extension torque at 180°/s	65	61	0.31 (−0.04, 0.66)	0.09	0
Timed walk over ground	72	71	−0.57 (−0.90, −0.24)	0.0008	0
Timed stair ascent and descent	72	71	−1.15 (−1.50, −0.79)	<0.00001	0
Timed walk over spongy surface	72	71	−1.66 (−2.05, −1.28)	<0.00001	0

*CG, control group; CI, confidence interval; IG, intervention group; SMD, standard mean difference*.

**Table 4:** Meta-analysis results of muscle strength and mobility when proprioceptive training compared to other non-proprioceptive training.

**Outcomes**	**IG (sample size)**	**CG (sample size)**	**SMD (95% CI)**	***P*-value**	***I*^**2**^ (%)**
Knee flexion torque at 60°/s	53	44	0.71 (0.30, 1.12)	0.0008	0
Knee extension torque at 60°/s	53	44	0.50 (−0.61, 1.61)	0.38	86
Timed walk over ground	72	71	0.06 (−0.28, 0.40)	0.72	6
Timed stair ascent and descent	72	71	0.35 (−0.09, 0.80)	0.12	44
Timed walk over spongy surface	72	71	−0.76 (−1.33, −0.18)	0.01	64
GUG test	30	27	−0.46 (−2.81, 1.89)	0.70	92

*CG, control group; CI, confidence interval; GUG, Get up and go; IG, intervention group; SMD, standard mean difference*.

##### Mobility

Two studies (57, 67) used the WST test to assess mobility. When proprioceptive training compared to no intervention, the subgroup analysis demonstrated that participants in the proprioceptive training group showed greater mobility when assessed for timed walk over ground (SMD = −0.57, 95%CI: [−0.90, −0.24], *I*^2^ = 0%, *P* = 0.0008), timed stair ascent and descent (SMD = −1.15, 95%CI: [−1.50, −0.79], *I*^2^ = 0%, *P* < 0.00001), and timed walk over spongy surface (SMD = −1.66, 95%CI: [−2.05,−1.28], *I*^2^ = 0%, *P* < 0.00001; Table 3). When proprioceptive training compared against other non-proprioceptive training, the meta-analysis revealed that participants in the proprioceptive training group presented increased mobility when assessed for timed walk over spongy surface (SMD = −0.76, 95%CI: [−1.33, −0.18], *I*^2^ = 0%, *P* = 0.01), but no statistically significant difference in timed walk over ground and timed stair ascent and descent (*P* > 0.05; Table 4). Furthermore, four studies (41, 58, 61, 62) used the GUG test to assess mobility. Subgroup analysis showed no statistically significant difference between the groups when proprioceptive training compared against other non-proprioceptive training (SMD = −0.46, 95%CI: [−2.81, 1.89], *I*^2^ = 92%, *P* = 0.70; Table 4). When proprioceptive training combined with other non-proprioceptive training compared against other non-proprioceptive training, the meta-analysis also revealed no significant difference in mobility (SMD = 0.05, 95%CI: [−0.55, 0.65], *I*^2^ = 46%, *P* = 0.87; Supplementary Figure S1).

##### Knee ROM and Balance

One study (45) used standard goniometric procedures (72) to measure knee ROM and showed significant improvement in knee ROM for proprioceptive training compared to no intervention (*P* < 0.05). In addition, when proprioceptive training plus other non-proprioceptive training compared against other non-proprioceptive training, Mondam et al. (63) used the Goniometer to measure knee ROM and demonstrated that there was statistically significantly greater knee ROM for the proprioceptive training plus other non-proprioceptive training group (*P* < 0.05). For balance, Gomiero et al. (52) assessed balance using the Tinetti balance scale and demonstrated that there was significant difference between the proprioceptive training group and the non-proprioceptive training group (*P* < 0.05).

##### Adverse Events

Only eight studies (41, 45, 52, 56, 58, 59, 64, 66) reported safety-related data, however some trials stated that no adverse events were reported (41, 64, 66), no serious adverse events during the intervention occurred (58). In addition, from another four studies (45, 52, 56, 58) that provided data (*n* = 210), 12 participants (5.7%) reported adverse events, including post-exercise soreness, back pain, hip soreness, foot pain, and ankle injury.

### Sensitivity Analysis

We used the leave-one-out method to conduct the sensitivity analyses for each of the evaluated outcomes (Supplementary Tables S3–S8). When proprioceptive training compared to other general non-proprioceptive training, the sensitivity analyses for WOMAC (total) score showed that the heterogeneity decreased to 0 after removing the study conducted by Vamsidhar et al. (68). When proprioceptive training plus conventional physiotherapy compared against conventional physiotherapy, the sensitivity analyses for pain and stiffness revealed that the heterogeneity decreased to 0 after removing Jahanjoo et al.'s study (44). And the sensitivity analyses for WOMAC (physical function) score showed that the removal of study conducted by Kumar et al. (54) significantly reduced the heterogeneity. The sensitivity analyses for WOMAC (total) score demonstrated that the removal of study conducted by Kirthika et al. (39) significantly reduced the heterogeneity. Furthermore, the sensitivity analyses for JPS showed the significant impact of proprioceptive training combined with conventional physiotherapy on JPS was highly affected by the study by Duman et al. (69). In general, these results suggested that these studies conducted by Vamsidhar et al. (68), Jahanjoo et al. (44), Kumar et al. (54), Kirthika et al. (39), or Duman et al. (69) could be the potential source of heterogeneity.

### Publication Bias

**Figure 7:**
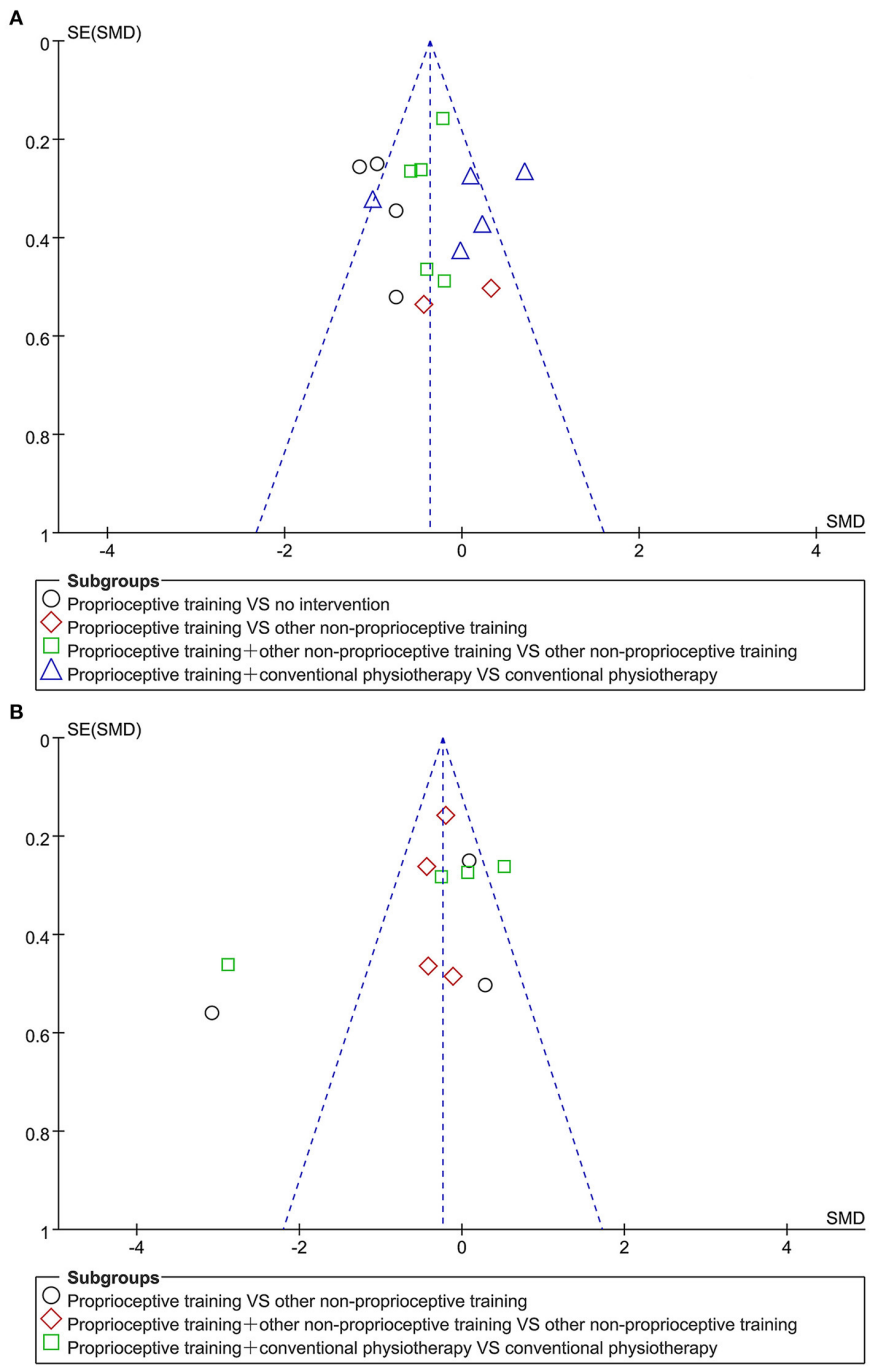
Funnel plots for publication bias assessment on WOMAC (physical function) score **(A)** and WOMAC (total) score **(B)**.

No obvious asymmetry was found by the visual inspection of funnel plots (Figure 7). There was no evidence for publication bias on the WOMAC (physical function) score (Begg's test, *P* = 0.56; Egger's regression, *P* = 0.95) and the WOMAC (total) score (Begg's test, *P* = 0.28; Egger's regression, *P* = 0.15).

### Quality of Evidence

Based on the GRADE approach for evaluating quality of the evidence, the results showed that there is moderate evidence in pain, low to moderate evidence in stiffness, physical function, muscle strength and mobility, and very low to moderate evidence in JPS. The corresponding information are detailed in Supplementary Table S3.

## Discussion

Results of this meta-analysis indicated that compared to no intervention, proprioceptive training significantly improved pain, stiffness, physical function, JPS, muscle strength, mobility, and knee ROM in people with KOA. When compared to other non-proprioceptive training, proprioceptive training presented similar outcomes, only providing greater results in terms of JPS and mobility (timed walk over spongy surface). When proprioceptive training plus other non-proprioceptive training compared to other non-proprioceptive training, the two groups showed similar outcomes, but there was a greater improvement for JPS, physical function, and knee ROM in the proprioceptive training plus other non-proprioceptive training group. When proprioceptive training combined with conventional physiotherapy compared against conventional physiotherapy, the two groups demonstrated similar outcomes, but there was a significant improvement for JPS in the proprioceptive training combined with conventional physiotherapy group. In addition, although only eight studies reported safety-related data, considering the low rate of reported adverse events (5.7%) and about half of the reported adverse events (*n* = 5) were normal reactions after exercise, such as post-exercise soreness. Therefore, proprioceptive training could still be considered a relatively safe intervention for the treatment of KOA.

In this present study, the outcome measurements of JPS and walking speed over spongy surfaces were also specifically used to assess proprioception. When proprioceptive training compared to no intervention or other non-proprioceptive training, participants in proprioceptive training group showed greater improvement in the two outcomes. Similarly, when proprioceptive training plus other non-proprioceptive training compared against other non-proprioceptive training or proprioceptive training combined with conventional physiotherapy compared against conventional physiotherapy, participants in the groups that included proprioceptive training presented significantly improved JPS. This further demonstrated that the proprioceptive training could specifically improve knee proprioception in patients with KOA, thereby indicating the effectiveness of this intervention.

We found that the measurements of the muscle strength/torque were inconsistent. Previous studies have indicated that the improvement of proprioception could promote the increase of muscle strength (73–75). The results were partially consistent with our findings. When proprioceptive training compared to other non-proprioceptive training, the intervention in the other non-proprioceptive training group was knee extension exercises. This may account for the finding that the greater improvement of knee extensor torque was observed in the other non-proprioceptive training group with respect to muscle strength of the knee extensors. In addition, we also found that there was a greater improvement for physical function and knee ROM in the proprioceptive training plus other non-proprioceptive training group. This may be due to the greater training intensity and frequency of the combined intervention, as well as the greater variety of training method.

### Comparison to Prior Reviews

To our knowledge, this is the first systematic review and meta-analysis to evaluate the safety of proprioceptive training for KOA and the effects of combinations of proprioceptive training with other interventions in KOA. In recent years, only a few studies have systematically investigated the effect of proprioceptive training on knee proprioception and function in KOA. Smith et al. (35) conducted a study to determine the effectiveness of proprioceptive training for KOA, the results indicated that significant improvement in functional outcomes (e.g., physical function, JPS, muscle strength, and mobility) for proprioceptive training compared to no intervention. When compared to other general non-proprioceptive training, proprioceptive training presented similar outcomes, only providing greater results in terms of JPS and mobility (timed walk over spongy surface). This is consistent with our review. In the most recent review (42), there is some evidence that proprioceptive training effectively improved pain and physical function in KOA, but stiffness and some other mobility measures (e.g., GUG) were unchanged after proprioceptive training. This is partially in line with our findings. The differences in the grouping of interventions may account for these inconsistent results.

### Limitations

However, this present study had several following limitations. Firstly, methodologic limitations (e.g., inadequately concealed allocation, a small number of studies using therapists/subjects blinding or assessors blinding, high dropout rates, and inconsistent use of “intention-to-treat” analyses) could overestimate the overall effect size. Secondly, given the relatively small number of studies included in our review and the low number of participants per study, some results cannot be considered robust. Thirdly, since some of the included studies did not describe the race or age of the participants, we could not conduct a subgroup analysis based on race or age. Additionally, due to the relatively small number of included studies, subgroup analysis based on intervention characteristics (e.g., duration, dose or intensity) could not be carried out. Therefore, we could not determine the influence of these factors on the results. Fourthly, in our study, the components of the proprioceptive training programs in each study are different. We should perform the meta-analysis on each of the proprioceptive training separately, however, this is not possible given the few studies there are. These factors may also have a potential influence on our results. Furthermore, only two studies assessed outcomes after a year of follow-up. Accordingly, it is unable to determine the long-term effects of proprioceptive training in KOA.

### Implications for Further Research and Practice

A larger and higher quality body of evidence is required before definite conclusions or recommendations could be made. Given that the main drawback of this review was the poor methodological quality of the included studies, future trials should use rigorous methodology to further ensure adequate concealed allocation, randomization, assessors blinding, and “intention-to-treat” analyses. Future researchers should improve the reporting in accordance with CONSORT guidelines (76). Future trials must also improve reporting of safety. Considering that this type of training may have potential therapeutic value, therefore it is necessary to further evaluate the optimal type of proprioceptive training. Additionally, the RCTs included in this review predominantly involved participants aged 50 years or over who presented early in their disease-stage. The majority of patients presented with moderate KOA (Kellgren-Lawrence grade II or III). Furthermore, the studies involving participants who had suffered knee joint trauma or surgery were also eliminated. Namely, this study only investigated patients with degenerative KOA. Given all this, the results of our study could only be applied to this KOA population. Therefore, it is still unclear whether the same clinical results would be obtained if proprioceptive training was prescribed for older people or those with more advanced KOA or those with KOA due to joint trauma or surgery. Further studies are needed to evaluate the clinical applicability of this exercise regime in different populations with knee OA.

## Conclusions

In conclusion, this present study indicated that proprioceptive training is safe and effective in treating KOA. There is some evidence that proprioceptive training combined with general non-proprioceptive training or conventional physiotherapy appears to be more effective and should be considered as part of the rehabilitation program. However, given that the majority of current studies investigated the short-term effect of these proprioceptive training program, more large-scale and well-designed studies with long-term follow up are needed to determine the long-term effects of these proprioceptive training regimes in KOA.

## Data Availability Statement

The datasets presented in this study can be found in online repositories. The names of the repository/repositories and accession number(s) can be found in the article/Supplementary Material.

## Author Contributions

YW and XX came up with the initial study design. YW, ZW, ZC, XY, and JY reviewed all of the studies, extracted the data, analyzed and interpreted the data, and drafted the manuscript. YW, ZW, YG, JW, and GC were involved in quality assessment. PZ, FX, WC, and ZY checked the data extraction and analysis for accuracy. XX critically revised the manuscript for important intellectual content. All authors have reviewed and approved the final version of the manuscript.

## Funding

This work was supported by Science and Technology Innovation Strategy Special Fund of Guangdong Province (2021B1111610007); Science and Technology Plan Project of Guangdong Province (2019A141401008); Soft Science Research Project of Guangdong Province (No. 2018B020207009); and Natural Science Foundation of Guangdong Province (2021A1515011545).

## Conflict of Interest

The authors declare that the research was conducted in the absence of any commercial or financial relationships that could be construed as a potential conflict of interest.

## Publisher's Note

All claims expressed in this article are solely those of the authors and do not necessarily represent those of their affiliated organizations, or those of the publisher, the editors and the reviewers. Any product that may be evaluated in this article, or claim that may be made by its manufacturer, is not guaranteed or endorsed by the publisher.
