# Supplementary material for: Proprioceptive Training for Knee Osteoarthritis: A Systematic Review and Meta-Analysis of Randomized Controlled Trials
Source: Front Med (Lausanne). 2021 Oct 28;8:699921. doi: 10.3389/fmed.2021.699921 (PMC8581183; doi:10.3389/fmed.2021.699921)
Supplement: Supplementary file 1 [file Data_Sheet_1.doc]

***Supplementary Materials***

Proprioceptive training for knee osteoarthritis: a systematic review and meta-analysis of randomized controlled trials

Included elements in this document: 1 Figure and 8 Tables

Figure S1- Forest plot of meta-analysis on mobility (GUG test)

Table S1- Search strategy

Table S2-Description of interventions used in the included studies

Table S3-8- Sensitivity analyses

Table S9- Evidence quality assessment according to GRADE.


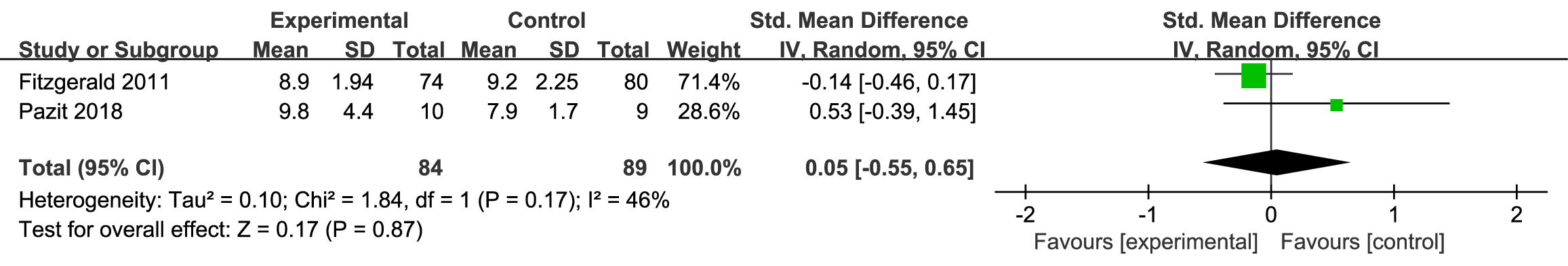


**FIGURE S1** Forest plot of meta-analysis on mobility (GUG test).

| **Table S1-Search strategy used** | |
| --- | --- |
| **PubMed** | #1 ("Osteoarthritis, Knee"[Mesh]) OR ((((Knee Osteoarthritides[Title/Abstract]) OR (Knee Osteoarthritis[Title/Abstract])) OR (Osteoarthritis of Knee[Title/  Abstract])) OR (Osteoarthritis of the Knee[Title/Abstract])) 26,576  #2 ((proprioception training) OR (("Circuit-Based Exercise"[Mesh]) OR ((((((  Circuit Based Exercise[Title/Abstract]) OR (Circuit-Based Exercises[Title/  Abstract])) OR (Exercise, Circuit-Based[Title/Abstract])) OR (Exercises, Circuit-Based[Title/Abstract])) OR (Circuit Training[Title/Abstract])) OR (Training,  Circuit[Title/Abstract] OR (balance training[Title/Abstract])))) OR (sensorimotor training)) 11,547  #3 (randomized controlled trial[pt] OR controlled clinical trial[pt] OR randomized[tiab] OR placebo[tiab] OR drug therapy[sh] OR randomly[tiab] OR trial[tiab] OR groups[tiab] NOT (animals [mh] NOT humans [mh])) 4,386,564  #4 #1 AND #2 AND #3 88 |
| **Embase** | #1 'knee osteoarthritis'/exp 38,340  #2 'arthrosis, knee':ab,ti OR 'femorotibial arthrosis':ab,ti OR 'gonarthrosis':ab,ti OR 'knee arthrosis':ab,ti OR 'knee joint arthrosis':ab,ti OR 'knee osteo-arthritis':ab,ti OR 'knee osteoarthritis':ab,ti 20,939 #3 #1 OR #2 38,849  #4 'proprioception training'/exp OR 'proprioception training':ab,ti OR 'balance training'/exp OR 'balance training':ab,ti OR 'sensorimotor training'/exp OR 'sensorimotor training':ab,ti 2,456 #5 'randomized controlled trial'/exp OR 'controlled clinical trial'/exp OR 'randomized':ti,ab OR 'placebo':ti,ab OR 'drug therapy':lnk OR 'randomly':ti,ab OR 'trial':ti,ab OR 'groups':ti,ab 7,818,309  **#**6 #3 AND #4 AND #5 32 |
| **Cochrane Library** | #1 MeSH descriptor: [Osteoarthritis, Knee] explode all trees 4,491  #2 (Osteoarthritis of Knee):ti,ab,kw OR (Osteoarthritis of the Knee):ti,ab,kw OR (Knee Osteoarthritis):ti,ab,kw OR (Knee Osteoarthritides):ti,ab,kw (Word variations have been searched) 12,936  #3 #1 OR #2  12,936  #4 (proprioception training):ti,ab,kw OR (balance training):ti,ab,kw OR (sensorimotor training):ti,ab,kw(Word variations have been searched) 9,245  #5  #3 AND #4 261 |
| **Web of**  **Science** | #1 TS= (Knee Osteoarthritis OR Osteoarthritis, Knee OR Knee Osteoarthritides OR Osteoarthritis of Knee OR Osteoarthritis of the Knee OR arthrosis, knee OR femorotibial arthrosis OR gonarthrosis OR knee arthrosis OR knee joint arthrosis OR knee joint osteoarthritis OR knee osteo-arthritis OR knee osteo-arthrosis OR knee osteoarthrosis OR osteoarthritis, knee OR osteoarthrosis, knee) 66,719  #2 TS= (proprioception training OR balance training OR sensorimotor training)  32,569  #3 TS=(randomized controlled trial OR randomized) 1,059,235  #4 #1 AND #2 AND #3 146 |

| **Table S2-Description of interventions used in the included studies** | |
| --- | --- |
| Reference | Intervention |
| Gomiero et al (2018) | Proprioceptive training: a program emphasizing agility, coordination and balance consisting of walking in different directions following verbal commands from the therapist; crossing steps while walking; crossing steps while walking back-wards; implementing sudden changes of direction; walking on several types of surfaces (including mattresses); maintaining posture during use of a balance board; and using a mini-trampoline to expose individuals to potentially destabilizing loads. |
|  | Resistance training: warm-up on a stationary bicycle for 10 minutes, quadriceps and hamstring strengthening exercises using ankle weights, isometric exercises for the quadriceps muscle (hip flexion with leg extended) and stretching for the lower limbs (stretching of the quadriceps, hamstrings and triceps surae) × 10 reps of maximal repetitions × 3 sets. All performed bilaterally. |
| Tsauo et al (2008) | Proprioceptive training: a sensorimotor training programme was used to facilitate joint position sense and dynamic joint stabilization using rhythmic active motion, angle repositioning and standing on an air cushion with support to stimulate muscular co activation. The equipment included: two sets of sling suspension systems, a metronome and an electrogoniometer. The programmes progressed from supine to a sitting position to a standing position to increase knee weight bearing. A sensory challenge was given by doing the exercises with eyes open or eyes closed in each position. |
|  | Conventional physiotherapy: thermotherapy, interferential therapy and instructions for exercise at home. Straight leg raising, short arc extension exercise, and walking or 30 min (within a tolerable time interval) were suggested as the home programme. |
| Kumar et al (2013) | Proprioceptive training: a multi-station exercise program consisting of 11 walking exercises. e.g., walk forward through 6 boxes (50cm × 50cm) on one-foot (in-in-out to right-in-in-out to left). Stair-up and -down a regular 3 steps staircase (17 cm high and 23 cm wide). Repeat the exercise 3 with hands behind the back. Followed by climbing a regular 3 steps staircase (17 cm high and 23 cm wide), -up and -down. |
|  | Conventional physiotherapy: ultrasound therapy (UST), warm up (walking or static bicycling and active stretching of knee, hip and ankle each for 2 min), open-chain exercises following the Delorme regimen of progressive resistive exercise × 10reps × 3 sets. |
| Fitzgerald et al (2011) | Proprioceptive training: received same standard exercise programme in addition of agility and perturbation exercises: side stepping, braiding (lateral stepping combined with forward and backward crossover steps), front crossover steps during forward ambulation, back crossover steps during backward ambulation, shuttle walking, walking with changes, tilt boards and roller boards. |
|  | Strength Training: lower limb muscle stretching (quadriceps, hamstring and calf muscle stretching) and strengthening (quadriceps sets, |
| **Table S2（continued）** | |
|  | supine straight leg raises, prone hip extensions, seated isometric knee extensions, single-leg leg presses, standing hamstring curls and standing heel raises), long-sitting knee flexion and extension range of motion and treadmill walking. |
| Rogers et al (2012) | Proprioceptive training: wedding march, backward wedding march, high knees march, side stepping, semi-tandem walk, tandem walk, cross-over walk, modified grapevine, toe walking, heel walking, static balance, dynamic balance. |
|  | Resistance training: ankle extension and flexion, knee extension and flexion, hip abduction and adduction, internal and external rotation, leg press. |
|  | Without intervention |
| Ahmed et al (2011) | Proprioceptive training: standing upright position (30 s) on a firm surface, then on a soft surface (a mat). single leg stance with closed eyes. forward stepping lunge, T-band kicks exercise, toe skipping, heel skipping, squatting exercise, balance exercise on wobble board. |
|  | Traditional exercise: range of motion and stretching exercises applied to hamstring and calf muscle, and quadriceps and hamstring isometric strengthening exercise. straight leg raising exercises, short-arc terminal extension exercise for the knee joint, and isometric exercises for the abductor and adductor muscles of the hip. short-arc terminal extension exercise with resistance for the knee joint, and isotonic strengthening exercise with resistance for the hamstring muscles. |
| Lin et al (2007) | Proprioceptive training: computer foot stepping games mainly involving knee movement in a sitting position (stepping on targets in multiple directions) × each 20 min × 10-min break. |
|  | Resistance training: closed chain knee extension exercises leg press (10 × 10 sets). |
|  | Without intervention |
| Jahanjoo et al (2019) | Proprioceptive training: following physical therapy and a 30-minute break, the patients underwent 1-hour balance training including postural stability, limits of stability and weight shift using the Biodex Balance System SD. |
|  | Conventional physiotherapy: all patients received 10 sessions of one-hour treatment including hot pack, ultrasound (US), transcutaneous electrical nerve stimulation (TENS) and exercise. |
| Lin et al (2009) | Proprioceptive training: computer foot stepping games mainly involving knee movement in a sitting position (stepping on targets in multiple directions) × each 20 min × 10-min break. |
|  | Strength Training: full knee extension using dynamometer cable × 6 reps (per set) × 4 sets. |
|  | Without intervention |
| **Table S2（continued）** | |
| Gohil and Shukla (2020） | Proprioceptive training: using biofeedback. stand on the Equiboard barefooted. a safety frame was placed around the board so that the patient can hold on it. Patients were made to play different types of games. The red dot (ball), in each game represent the angular position of the Equiboard and the patient has to maintain his balance with the feedback position of the ball. |
|  | Conventional physiotherapy: quadriceps setting exercise, short arc terminal knee extension, straight leg raise, hip abductor strengthening in side lying, hamstring curls in prone lying, quadriceps strengthening in high sitting, hamstring and gastrocnemius muscle stretching, close chain exercise- partial squats and short wave diathermy. All exercises are performed thrice daily and 10 repetition of each exercise. |
| Kirthika et al (2018) | Proprioceptive training: Pelvic tilts and knee flexion and extension using Swiss ball, sliding lunge, stepping lunge, step up and down in a footstool, stand up and sit down in a stool, squatting, and single leg squat were given as proprioceptive exercises. All the exercises were performed once in a day, with 10 repetitions for each exercise. |
|  | Conventional physiotherapy: isometric quadriceps exercises, high sitting knee extension, straight leg raise, hamstring stretching, hip abduction, hip extension, along with short‑wave diathermy (crossfire method) wider spacing, and thermal dose for 15 min. All the exercises were performed once in a day, with 10 repetitions for each exercise. |
| Pzait et al (2018) | Proprioceptive training: six balance exercises (walking forward and backward, single leg standing, single leg tapping, side stepping and backward walking) |
|  | Resistance training: consist of 6-8 exercises targeting the lower limbs (leg press, sit to stand, squat, step-up, calf raises lunges, going up stairs) supervised by a qualified Exercise Physiologist. |
| Takacs et al (2017) | Proprioceptive training: targeted dynamic balance training consisting of progressive exercise training over three phases. One phase: sitting rotation, chair sit/squat, calf raise, side stepping, stepping pattern. Two phase: standing rotation, step down, toe walking, lateral step-up, stepping pattern. Three phase: stepping rotation, lunge, mini-hop, skate stepping, cone walking. |
|  | Without intervention |
| Sekir and Gür (2005) | Proprioceptive training: a multi-station exercise program consisting of 11 walking exercises: walk forward through 6 boxes (50cm × 50cm) on one-foot (in-in-out to right-in-in-out to left). Stair-up and -down a regular 3 steps staircase (17 cm high and 23 cm wide). Repeat the exercise 3 with hands behind the back. Followed by climbing a regular 3 steps staircase (17 cm high and 23 cm wide), -up and -down. |
|  | Without intervention |
| **Table S2（continued）** | |
| Rogers et al (2011) | Proprioceptive training: wedding march, backward wedding march, high knees march, side stepping, semi-tandem walk, tandem walk, cross-over walk, modified grapevine, toe walking, heel walking. |
|  | Strength training: seated resistance band exercises: ankle extension, ankle flexion, knee extension, knee flexion, hip abduction, hip adduction, hip internal rotation, hip external rotation, leg press (hip and knee extension) other: standing hip hyper-extension with resistance band; standing wall slides (partial squats) with a small “play ball” behind the back; supine heel slides (hip and knee flexion and extension). |
| Chaipinyo and  Karoonsupcharoen  (2009) | Proprioceptive training: forward, backward, and sideward step ×30reps, bilateral mini squat ×10 reps. |
|  | Strength training: Isometric knee-extension exercise × 10 reps (per set) × 5s hold × 3 sets. |
| Mondam et al (2012) | Proprioceptive training: one leg balance, blind advanced one leg balance, toe walking, heel walking, cross body leg swings. |
|  | Conventional physiotherapy: Isometric quadriceps exercises were given with participants in long sitting position with hands at side, with a role of towel placed below affected knee. The subject was now asked to press the towel down and hold it till count of ten and then relax and repeat again for 10 repetitions followed by other side knee. |
| Dıracoglu et al (2005) | Proprioceptive training: progressive kinaesthesia, balance and strengthening exercises including modified Rhomberg exercise, retrowalking, walking on heels/toes, rocker board, one leg standing. |
|  | Strength training: static exercise bike, isometric hamstrings, quadriceps and abductor (6 s hold ×8), through range isotonic resisted quadriceps and hamstring exercises ×10 reps of maximal weight ×1 set. |
| Elgendy et al (2005) | Proprioceptive training: foot fits, anteroposterior rolling movement, sliding rolling movement, multidirectional rolling movement from sitting and multidirectional rolling movement from standing. |
|  | Traditional exercise: stretching the hamstring muscle, stretching the calf muscles straight leg raise exercise and isometric strengthening of the quadriceps muscle. |
| Song et al (2020) | Proprioceptive training: proprioceptive neuromuscular facilitation (PNF) stretching sessions included a 5-minute warm-up, 45-minute stretching, and 10-minute cooldown. The stretching included four movement patterns: flexion abduction-internal, extension-adduction-external, flexion-adduction-external, and extension-abduction-internal rotations. Each stretching pattern × 5-8 reps |
| **Table S2（continued）** | |
|  | (per set) ×3 sets. |
| Dıracoglu et al (2008) | Proprioceptive training: progressive kinaesthesia, balance and strengthening exercises including modified Rhomberg exercise, retrowalking, walking on heels/toes, rocker board, one leg standing. |
|  | Strength Training: static exercise bike, isometric hamstrings, quadriceps and abductor (6 s hold ×8), through range isotonic resisted quadriceps and hamstring exercises ×10 reps of maximal weight ×1 set. |
| Jan et al (2009) | Proprioceptive training: a virtual foot stepping exercise which is a target matching task. For each patient, each lower extremity was trained for 20 min, with a 10-minute break between lower extremities to prevent fatigue. |
|  | Resistance training: a pad (attached to the cable of the isotonic dynamometer) was placed on the anterior aspect of the distal lower leg. Participants were asked to extend the knee joint from 90° of knee flexion to full extension and then to flex the knee joint to the starting position with eccentric contraction of quadriceps at a speed of 90°/2s. |
|  | Without intervention |
| Vamsidhar et al (2017) | Proprioceptive training: one leg balance, blind advanced one leg balance, toe walking, heel walking, cross leg body swing. |
|  | Strength training: seated leg press, partial squats. |
| Duman et al (2012) | Proprioceptive training: quadriceps, ankle extension, hip abductor, bicycling, walking by making a 458 corner at every 2 steps (zigzag), walking forward and backward by heel to toe, walking sidelong to the right and left. |
|  | Conventional physiotherapy: infrared and shortwave therapy |
|  | NSAID meloxicam 15 mg/d |
| rep, repetition; NSAID, nonsteroidal anti-inflammatory drug | |

**Proprioceptive training VS. other non-proprioceptive training**

**Table S3-Sensitivity analyses for WOMAC (total) score**

| References | SMD (95%CI) | *P* | *I*^2^(%) |
| --- | --- | --- | --- |
| Gomiero2018 | -1.38 (-4.66, 1.90) | 0.41 | 95 |
| Rogers2012 | -1.45 (-4.54, 1.64) | 0.36 | 96 |
| Vamsidhar2017 | 0.13 (-0.31, 0.57) | 0.57 | 0 |
| CI, confidence interval; SMD, standard mean difference | | | |

**Proprioceptive training with conventional physiotherapy VS. conventional physiotherapy**

**Table S4-Sensitivity analyses for pain**

| References | SMD (95%CI) | *P* | *I*^2^(%) |
| --- | --- | --- | --- |
| Duman2012 | -0.54 (-1.42, 0.34) | 0.23 | 74 |
| Jahanjoo2019 | -0.08 (-0.51, 0.36) | 0.73 | 0 |
| Tsauo2008 | -0.52 (-1.36, 0.32) | 0.23 | 80 |
| CI, confidence interval; SMD, standard mean difference | | | |

| **Table S5-Sensitivity analyses for stiffness** | | | |
| --- | --- | --- | --- |
| References | SMD (95%CI) | *P* | *I*^2^(%) |
| Duman2012 | 0.50 (-0.41, 1.42) | 0.28 | 76 |
| Jahanjoo2019 | -0.04 (-0.47, 0.39) | 0.85 | 0 |
| Tsauo2008 | 0.44 (-0.54, 1.42) | 0.38 | 85 |
| CI, confidence interval; SMD, standard mean difference | | | |

| **Table S6-Sensitivity analyses for WOMAC (physical function) score** | | | |
| --- | --- | --- | --- |
| References | SMD (95%CI) | *P* | *I*^2^(%) |
| Duman2012 | -0.01 (-0.81, 0.78) | 0.97 | 82 |
| Gohil2020 | 0.02 (-0.70, 0.73) | 0.97 | 82 |
| Jahanjoo2019 | -0.18 (-0.77, 0.40) | 0.54 | 67 |
| Kumar2013 | 0.31 (-0.02, 0.64) | 0.06 | 11 |
| Tsauo2008 | -0.04 (-0.77, 0.69) | 0.91 | 82 |
| CI, confidence interval; SMD, standard mean difference | | | |

| **Table S7-Sensitivity analyses for WOMAC (total) score** | | | |
| --- | --- | --- | --- |
| References | SMD (95%CI) | *P* | *I*^2^(%) |
| Duman2012 | -0.82 (-2.46, 0.82) | 0.33 | 95 |
| Jahanjoo2019 | -0.97 (-2.43, 0.50) | 0.20 | 94 |
| Kirthika2018 | 0.12 (-0.31, 0.56) | 0.58 | 50 |
| Mondam2012 | -0.71 (-2.36, 0.93) | 0.39 | 95 |
| CI, confidence interval; SMD, standard mean difference | | | |

| **Table S8-Sensitivity analyses for JPS** | | | |
| --- | --- | --- | --- |
| References | SMD (95%CI) | *P* | *I*^2^(%) |
| Duman2012 | -1.25 (-1.78, -0.73) | ＜0.00001 | 24 |
| Gohil2020 | -0.68 (-1.41, 0.05) | 0.07 | 74 |
| Kumar2013 | -0.94 (-2.03, 0.16) | 0.09 | 84 |
| Tsauo2008 | -0.97 (-2.03, 0.09) | 0.07 | 85 |
| CI, confidence interval; SMD, standard mean difference | | | |

**Table S9-Evidence quality assessment according to GRADE.**

| **Proprioceptive training for knee osteoarthritis** | | | | | | |
| --- | --- | --- | --- | --- | --- | --- |
| **Patient or population:** patients with knee osteoarthritis **Settings:**  **Intervention:** Proprioceptive training or proprioceptive training combined with other interventions | | | | | | |
| **Outcomes** | **Illustrative comparative risks* (95% CI)** | | **Relative effect (95% CI)** | **No of Participants (studies)** | **Quality of the evidence (GRADE)** | **Comments** |
|  | Assumed risk | Corresponding risk |  |  |  |  |
|  | **Control** | **Proprioceptive training** |  |  |  |  |
| **Pain (Proprioceptive training VS no intervention)** |  | The mean pain (proprioceptive training vs no intervention) in the intervention groups was **1.07 standard deviations lower** (1.46 to 0.68 lower) |  | 117 (3 studies) | ⊕⊕⊕⊝ **moderate**^1^ | This is a moderate effect that is clinically relevant in this patient group |
| **Pain (Proprioceptive training VS other non-proprioceptive training)** |  | The mean pain (proprioceptive training vs other non-proprioceptive training) in the intervention groups was **0.02 standard deviations lower** (0.74 lower to 0.69 higher) |  | 31 (2 studies) | ⊕⊕⊕⊝ **moderate**^1^ | This is a moderate effect that is clinically relevant in this patient group |
| **Pain (Proprioceptive training plus other non-proprioceptive training VS other non-proprioceptive training)** |  | The mean pain (proprioceptive training plus other non-proprioceptive training vs other non-proprioceptive training) in the intervention groups was **0.17 standard deviations lower** (0.58 lower to 0.23 higher) |  | 96 (3 studies) | ⊕⊕⊕⊝ **moderate**^1^ | This is a moderate effect that is clinically relevant in this patient group |
| **Pain (Proprioceptive training plus conventional physiotherapy VS conventional physiotherapy)** |  | The mean pain (proprioceptive training plus conventional physiotherapy vs conventional physiotherapy) in the intervention groups was **0.39 standard deviations lower** (0.99 lower to 0.22 higher) |  | 143 (3 studies) | ⊕⊕⊕⊝ **moderate**^1^ | This is a moderate effect that is clinically relevant in this patient group |
| **Stiffness (Proprioceptive training VS other non-proprioceptive training)** |  | The mean stiffness (proprioceptive training vs other non-proprioceptive training) in the intervention groups was **0.06 standard deviations lower** (0.78 lower to 0.65 higher) |  | 31 (2 studies) | ⊕⊕⊕⊝ **moderate**^1^ | This is a moderate effect that is clinically relevant in this patient group |
| **Stiffness (Proprioceptive training plus other non-proprioceptive training VS other non-proprioceptive training)** |  | The mean stiffness (proprioceptive training plus other non-proprioceptive training vs other non-proprioceptive training) in the intervention groups was **0.09 standard deviations lower** (0.69 lower to 0.5 higher) |  | 96 (3 studies) | ⊕⊕⊝⊝ **low**^1,2^ | This is a small effect that may be clinically relevant in this patient group |
| **Stiffness (Proprioceptive training plus conventional physiotherapy VS conventional physiotherapy)** |  | The mean stiffness (proprioceptive training plus conventional physiotherapy vs conventional physiotherapy) in the intervention groups was **0.31 standard deviations higher** (0.37 lower to 0.99 higher) |  | 143 (3 studies) | ⊕⊕⊝⊝ **low**^1,3^ | This is a small effect that may be clinically relevant in this patient group |
| **WOMAC (physical function) score (Proprioceptive training VS no intervention)** |  | The mean WOMAC (physical function) score (proprioceptive training vs no intervention) in the intervention groups was **0.97 standard deviations lower** (1.26 to 0.67 lower) |  | 195 (4 studies) | ⊕⊕⊕⊝ **moderate**^1^ | This is a moderate effect that is clinically relevant in this patient group |
| **WOMAC (physical function) score (Proprioceptive training VS other non-proprioceptive training)** |  | The mean WOMAC (physical function) score (proprioceptive training vs other non-proprioceptive training) in the intervention groups was **0.03 standard deviations lower** (0.76 lower to 0.7 higher) |  | 31 (2 studies) | ⊕⊕⊕⊝ **moderate**^1^ | This is a moderate effect that is clinically relevant in this patient group |
| **WOMAC (physical function) score (Proprioceptive training plus other non-proprioceptive training VS other non-proprioceptive training)** |  | The mean WOMAC (physical function) score (proprioceptive training plus other non-proprioceptive training vs other non-proprioceptive training) in the intervention groups was **0.34 standard deviations lower** (0.56 to 0.12 lower) |  | 315 (5 studies) | ⊕⊕⊕⊝ **moderate**^1^ | This is a moderate effect that is clinically relevant in this patient group |
| **WOMAC (physical function) score (Proprioceptive training plus conventional physiotherapy VS conventional physiotherapy)** |  | The mean WOMAC (physical function) score (proprioceptive training plus conventional physiotherapy vs conventional physiotherapy) in the intervention groups was **0.01 standard deviations higher** (0.57 lower to 0.6 higher) |  | 209 (5 studies) | ⊕⊕⊝⊝ **low**^1,3^ | This is a small effect that may be clinically relevant in this patient group |
| **WOMAC (total) score (Proprioceptive training VS other non-proprioceptive training)** |  | The mean WOMAC (total) score (proprioceptive training vs other non-proprioceptive training) in the intervention groups was **0.86 standard deviations lower** (2.68 lower to 0.97 higher) |  | 110 (3 studies) | ⊕⊕⊝⊝ **low**^1,3^ | This is a small effect that may be clinically relevant in this patient group |
| **WOMAC (total) score (Proprioceptive training plus other non-proprioceptive training VS other non-proprioceptive training)** |  | The mean WOMAC (total) score (proprioceptive training plus other non-proprioceptive training vs other non-proprioceptive training) in the intervention groups was **0.26 standard deviations lower** (0.51 to 0.01 lower) |  | 255 (4 studies) | ⊕⊕⊕⊝ **moderate**^1^ | This is a moderate effect that is clinically relevant in this patient group |
| **WOMAC (total) score (Proprioceptive training plus conventional physiotherapy VS conventional physiotherapy)** |  | The mean WOMAC (total) score (proprioceptive training plus conventional physiotherapy vs conventional physiotherapy) in the intervention groups was **0.57 standard deviations lower** (1.69 lower to 0.54 higher) |  | 204 (4 studies) | ⊕⊕⊝⊝ **low**^1,3^ | This is a small effect that may be clinically relevant in this patient group |
| **JPS (Proprioceptive training VS no intervention)** |  | The mean JPS (proprioceptive training vs no intervention) in the intervention groups was **1.73 standard deviations lower** (2.09 to 1.37 lower) |  | 165 (3 studies) | ⊕⊕⊕⊝ **moderate**^1,4^ | This is a moderate effect that is clinically relevant in this patient group |
| **JPS (Proprioceptive training VS other non-proprioceptive training)** |  | The mean JPS (proprioceptive training vs other non-proprioceptive training) in the intervention groups was **1.28 standard deviations lower** (1.64 to 0.92 lower) |  | 143 (2 studies) | ⊕⊕⊕⊝ **moderate**^1,4^ | This is a moderate effect that is clinically relevant in this patient group |
| **JPS (Proprioceptive training plus other non-proprioceptive training VS other non-proprioceptive training)** |  | The mean JPS (proprioceptive training plus other non-proprioceptive training vs other non-proprioceptive training) in the intervention groups was **1.54 standard deviations lower** (2.74 to 0.34 lower) |  | 70 (2 studies) | ⊕⊝⊝⊝ **very low**^1,3,4^ | This is a weak effect that is clinically relevant in this patient group |
| **JPS (Proprioceptive training plus conventional physiotherapy VS conventional physiotherapy)** |  | The mean JPS (proprioceptive training plus conventional physiotherapy vs conventional physiotherapy) in the intervention groups was **0.95 standard deviations lower** (1.73 to 0.18 lower) |  | 149 (4 studies) | ⊕⊝⊝⊝ **very low**^1,3,4^ | This is a weak effect that is clinically relevant in this patient group |
| **Muscle strength-Knee flexion torque (60°/s) (Proprioceptive training VS no intervention)** |  | The mean muscle strength-knee flexion torque (60°/s) (proprioceptive training vs no intervention) in the intervention groups was **0.65 standard deviations higher** (0.29 to 1.01 higher) |  | 126 (2 studies) | ⊕⊕⊕⊝ **moderate**^1^ | This is a moderate effect that is clinically relevant in this patient group |
| **Muscle strength-Knee flexion torque (60°/s) (Proprioceptive training VS other non-proprioceptive training)** |  | The mean muscle strength-knee flexion torque (60°/s) (proprioceptive training vs other non-proprioceptive training) in the intervention groups was **0.71 standard deviations higher** (0.3 to 1.12 higher) |  | 97 (2 studies) | ⊕⊕⊕⊝ **moderate**^1^ | This is a moderate effect that is clinically relevant in this patient group |
| **Muscle strength-Knee flexion torque (120°/s) (Proprioceptive training VS no intervention)** |  | The mean muscle strength-knee flexion torque (120°/s) (proprioceptive training vs no intervention) in the intervention groups was **0.32 standard deviations higher** (0.03 lower to 0.67 higher) |  | 126 (2 studies) | ⊕⊕⊕⊝ **moderate**^1^ | This is a moderate effect that is clinically relevant in this patient group |
| **Muscle strength-Knee flexion torque (180°/s) (Proprioceptive training VS no intervention)** |  | The mean muscle strength-knee flexion torque (180°/s) (proprioceptive training vs no intervention) in the intervention groups was **0.25 standard deviations higher** (0.1 lower to 0.6 higher) |  | 126 (2 studies) | ⊕⊕⊕⊝ **moderate**^1^ | This is a moderate effect that is clinically relevant in this patient group |
| **Muscle strength-Knee extension torque (60°/s) (Proprioceptive training VS no intervention)** |  | The mean muscle strength-knee extension torque (60°/s) (proprioceptive training vs no intervention) in the intervention groups was **0.42 standard deviations higher** (0.07 to 0.78 higher) |  | 126 (2 studies) | ⊕⊕⊕⊝ **moderate**^1^ | This is a moderate effect that is clinically relevant in this patient group |
| **Muscle strength-Knee extension torque (60°/s) (Proprioceptive training VS other non-proprioceptive training)** |  | The mean muscle strength-knee extension torque (60°/s) (proprioceptive training vs other non-proprioceptive training) in the intervention groups was **0.5 standard deviations higher** (0.61 lower to 1.61 higher) |  | 97 (2 studies) | ⊕⊕⊝⊝ **low**^1,3^ | This is a small effect that may be clinically relevant in this patient group |
| **Muscle strength-Knee extension torque (120°/s) (Proprioceptive training VS no intervention)** |  | The mean muscle strength-knee extension torque (120°/s) (proprioceptive training vs no intervention) in the intervention groups was **0.3 standard deviations higher** (0.05 lower to 0.65 higher) |  | 126 (2 studies) | ⊕⊕⊕⊝ **moderate**^1^ | This is a moderate effect that is clinically relevant in this patient group |
| **Muscle strength-Knee extension torque (180°/s) (Proprioceptive training VS no intervention)** |  | The mean muscle strength-knee extension torque (180°/s) (proprioceptive training vs no intervention) in the intervention groups was **0.31 standard deviations higher** (0.04 lower to 0.66 higher) |  | 126 (2 studies) | ⊕⊕⊕⊝ **moderate**^1^ | This is a moderate effect that is clinically relevant in this patient group |
| **Mobility-Timed walk over ground (Proprioceptive training VS no intervention)** |  | The mean mobility-timed walk over ground (proprioceptive training vs no intervention) in the intervention groups was **0.57 standard deviations lower** (0.9 to 0.24 lower) |  | 143 (2 studies) | ⊕⊕⊕⊝ **moderate**^1^ | This is a moderate effect that is clinically relevant in this patient group |
| **Mobility-Timed walk over ground (Proprioceptive training VS other non-proprioceptive training)** |  | The mean mobility-timed walk over ground (proprioceptive training vs other non-proprioceptive training) in the intervention groups was **0.06 standard deviations higher** (0.28 lower to 0.4 higher) |  | 143 (2 studies) | ⊕⊕⊕⊝ **moderate**^1^ | This is a moderate effect that is clinically relevant in this patient group |
| **Mobility-Timed stair ascent and descent (Proprioceptive training VS no intervention)** |  | The mean mobility-timed stair ascent and descent (proprioceptive training vs no intervention) in the intervention groups was **1.15 standard deviations lower** (1.5 to 0.79 lower) |  | 143 (2 studies) | ⊕⊕⊕⊝ **moderate**^1^ | This is a moderate effect that is clinically relevant in this patient group |
| **Mobility-Timed stair ascent and descent (Proprioceptive training VS other non-proprioceptive training)** |  | The mean mobility-timed stair ascent and descent (proprioceptive training vs other non-proprioceptive training) in the intervention groups was **0.35 standard deviations higher** (0.09 lower to 0.8 higher) |  | 143 (2 studies) | ⊕⊕⊝⊝ **low**^1,2^ | This is a small effect that may be clinically relevant in this patient group |
| **Mobility-Timed walk over spongy surface (Proprioceptive training VS no intervention)** |  | The mean mobility-timed walk over spongy surface (proprioceptive training vs no intervention) in the intervention groups was **1.66 standard deviations lower** (2.05 to 1.28 lower) |  | 143 (2 studies) | ⊕⊕⊕⊝ **moderate**^1^ | This is a moderate effect that is clinically relevant in this patient group |
| **Mobility-Timed walk over spongy surface (Proprioceptive training VS other non-proprioceptive training)** |  | The mean mobility-timed walk over spongy surface (proprioceptive training vs other non-proprioceptive training) in the intervention groups was **0.76 standard deviations lower** (1.33 to 0.18 lower) |  | 143 (2 studies) | ⊕⊕⊝⊝ **low**^1,3^ | This is a small effect that may be clinically relevant in this patient group |
| **Mobility-GUG test (Proprioceptive training VS other non-proprioceptive training)** |  | The mean mobility-GUG test (proprioceptive training vs other non-proprioceptive training) in the intervention groups was **0.46 standard deviations lower** (2.81 lower to 1.89 higher) |  | 57 (2 studies) | ⊕⊕⊕⊝ **moderate**^1^ | This is a moderate effect that is clinically relevant in this patient group |
| **Mobility-GUG test (Proprioceptive training plus other non-proprioceptive training VS other non-proprioceptive training)** |  | The mean mobility-GUG test (proprioceptive training plus other non-proprioceptive training vs other non-proprioceptive training) in the intervention groups was **0.05 standard deviations higher** (0.55 lower to 0.65 higher) |  | 173 (2 studies) | ⊕⊕⊝⊝ **low**^1,3^ | This is a small effect that may be clinically relevant in this patient group |
| **Adverse events** | See comment | See comment | Not estimable | 210 (8 studies) | See comment | Eight studies reported adverse events, but no complete data were available |
| *The basis for the **assumed risk** (e.g. the median control group risk across studies) is provided in footnotes. The **corresponding risk** (and its 95% confidence interval) is based on the assumed risk in the comparison group and the **relative effect** of the intervention (and its 95% CI).  **CI:** Confidence interval; | | | | | | |
| GRADE Working Group grades of evidence **High quality:** Further research is very unlikely to change our confidence in the estimate of effect.  **Moderate quality:** Further research is likely to have an important impact on our confidence in the estimate of effect and may change the estimate. **Low quality:** Further research is very likely to have an important impact on our confidence in the estimate of effect and is likely to change the estimate. **Very low quality:** We are very uncertain about the estimate. | | | | | | |
| ^1^ Downgraded one level due to imprecision (fewer than 400 participants, total). ^2^ Downgraded one level due to clear inconsistency of results. ^3^ Downgraded one level due to inconsistency (I² > 50%). ^4^ Downgraded one level due to different measurement method. | | | | | | |
